# Review of the East Palaearctic and North Oriental *Psyttalia* Walker, with the description of three new species (Hymenoptera, Braconidae, Opiinae)

**DOI:** 10.3897/zookeys.629.10167

**Published:** 2016-11-07

**Authors:** Qiong Wu, Cornelis van Achterberg, Jiang-Li Tan, Xue-Xin Chen

**Affiliations:** 1 Institute of Insect Sciences, Zhejiang University, Zijingang Campus, Yuhangtang Road 866, Hangzhou 310058, China; 2 Shaanxi Key Laboratory for Animal Conservation, Northwest University, 229 North Taibai Road, Xi’an, Shaanxi 710069, China

**Keywords:** Braconidae, Opiinae, Psyttalia, new species, Tephritidae, East Palaearctic, North Oriental, Japan, China, Far East Russia, Korea, Netherlands, Norway

## Abstract

The East Palaearctic and North Oriental species of the genus *Psyttalia* Walker (Hymenoptera, Braconidae, Opiinae) are reviewed. Three new species are described and illustrated: *Psyttalia latinervis* Wu & van Achterberg, **sp. n**. and *Psyttalia majocellata* Wu & van Achterberg, **sp. n**. from China, and *Psyttalia spectabilis* van Achterberg, **sp. n.** from Japan. *Coeloreuteus formosanus* Watanabe, 1934, Opius (Lissosema) proclivis Papp, 1981, Opius (Psyttalia) subcyclogaster Tobias, 1998, Opius (Psyttalia) darasunicus Tobias, 1998, Opius (Psyttalia) cyclogastroides Tobias, 1998, *Psyttalia extensa* Weng & Chen, 2001, and *Rhogadopsis longicaudifera* Li & van Achterberg, 2013, are new synonyms of *Psyttalia cyclogaster* (Thomson, 1895); Opius (Psyttalia) ophthalmicus Tobias, 1977, and Opius (Psyttalia) brevitemporalis Tobias, 1998, of *Psyttalia carinata* (Thomson, 1895) and both Opius (Psyttalia) vacuus Tobias, 1998, and Opius (Lissosema) longurius Chen & Weng, 1995, of *Rhogadopsis mediocarinata* (Fischer, 1963). *Phaedrotoma daghestanicum* (Telenga, 1950), *Rhogadopsis mediocarinata* (Fischer, 1963) and *Rhogadopsis mystica* (Fischer, 1963) are new combinations. New records are *Psyttalia carinata* (Thomson, 1895) from The Netherlands and Norway, and *Psyttalia cyclogaster* (Thomson, 1895) from Japan. A lectotype is designated for *Psyttalia carinata* (Thomson, 1895) and *Psyttalia cyclogaster* (Thomson, 1895). A key to the East Palaearctic and North Oriental species of the genus *Psyttalia* Walker is included.

## Introduction

The large subfamily Opiinae (Braconidae), with 2,020+ valid species (Yu et al. 2012, van Achterberg et al. 2012, Li et al. 2013), is a common group of generally small (2–5 mm) parasitoid wasps. It has a worldwide distribution and the world fauna has been reviewed by Fischer (1972, 1977, 1986, 1987). Wharton (1988, 1997), van Achterberg (1997, 2004a, 2004b), van Achterberg and Salvo (1997), van Achterberg and Chen (2004) and Li et al. (2013) published updates or some additions for the existing keys to the genera of the Opiinae, but the number of genera and the limits of several genera are still matter of discussion. Currently about 39 genera are used, with about 60 additional names circulating in the existing literature; mostly as subgenera in the genus *Opius* Wesmael s.l. Recently, 28 subgenera were synonymized by Li et al. (2013).

*Psyttalia* is a fairly large genus, currently with 79 valid species (Wharton 2009). The number of valid species in the Palaearctic and Oriental regions is unknown because of undercollecting and different generic limits used by different authors. Several of the species listed by Wharton (2009) after examination of the types proved to be junior synonyms or belong to other genera (e.g. *Psyttalia vacua*; see below). Nevertheless, the total number will be much more than 80, because several undescribed species are recognised in existing collections (e.g. Wharton 2009 and personal experience of authors) and cryptic species are likely present (Wharton 2009). Fischer (1972, 1987) and Wharton (2009) divided the species into two main groups (A: vein m-cu of fore wing antefurcal or interstitial; B: vein m-cu postfurcal) but this is problematical and too simplistic. For instance, *Psyttalia cyclogaster* has either vein m-cu distinctly postfurcal (group B; Figs 13–14) or subinterstitial (group A).

Opiinae are solitary koinobiont endoparasitoids of larvae of cyclorraphous Diptera, but oviposition may take place in the egg of the host (ovo-larval parasitoids). The parasitoid larva has its final development when the host larva has made its puparium and the adult wasp emerges from this puparium. Opiinae may play an important role in the biocontrol of dipterous pests as fruit-infesting Tephritidae and mining Agromyzidae and the genus *Psyttalia* is no exception. Several species (e.g. *Psyttalia fletcheri*, *Psyttalia incisi*, *Psyttalia makii*) have been introduced to control fruit flies (Wharton 2009, Yu et al. 2012) with variable success.

## Material and methods

The material examined is deposited in the collections of the Zhejiang University (ZJUH) at Hangzhou, Northwest University (NWUX) at Xi’an, Institute of Zoology (IZAS) at Beijing, Naturalis Biodiversity Center (RMNH) at Leiden, Hungarian National Museum for Natural History (MTMA) at Budapest and Zoological Institute (ZISP) at St. Petersburg. The specimens collected by the third author during fieldwork on the Qinling Mts in Shaanxi province (Northwest China) and the type series of *Psyttalia spectabilis* were directly preserved in alcohol and the specimens were later prepared with the AXA method (van Achterberg 2009), the other specimens were collected by hand net and later card-pointed.

For identification of the subfamily Opiinae, see van Achterberg (1990, 1993), for identification of the genus, see Wharton (1997, 2009), Chen and Weng (2005) and the diagnosis in this paper. Wharton’s (1987, 1997, 2009) interpretation of the genus is followed here; only a combination of the listed characters allows a valid identification because of the observed variation in most characters and the less variable characters are not exclusive for the genus (Wharton 2009). For references to the biology, see Yu et al. (2012) and for the terminology used in this paper, see van Achterberg (1988, 1993). Measurements are taken as indicated by van Achterberg (1988). Morphological terminology follows van Achterberg (1988, 1993), including the abbreviations for the wing venation. Measurements are taken as indicated by van Achterberg (1988): for the length and the width of a body part the maximum length and width is taken, unless otherwise indicated. The length of the mesosoma is measured from the anterior border of the mesoscutum till the apex of the propodeum and of the first tergite from the posterior border of the adductor till the medio-posterior margin of the tergite. A new provincial record of China is indicated by an asterisk.

Descriptions and measurements were made under a stereomicroscope (Zeiss Stemi SV 6). Photographs were made with an Olympus SZX12 motorized stereomicroscope with AnalySIS Extended Focal Imaging Software or with Keyence VHX-2000 and -5000 digital microscopes. Adobe Photoshop software was used to make small adjustments and to assemble the plates.

## Results

### Psyttalia

Taxon classificationAnimaliaHymenopteraBraconidae

Walker, 1860

Figs 1 , 2–12 , 13 , 14–24 , 25–27 , 28–32 , 33 , 34–43 , 44 , 45–52 , 53 , 54–64 , 65 , 66–76 , 77 , 78–88 , 89 , 90–99 , 100 , 101–110

Psyttalia Walker, 1860: 311. Type species (by monotypy): Psyttalia testacea Walker, 1860 (= Opius walkeri Muesebeck, 1931) [examined].
Mesostoma Cameron, 1905: 42. Type species (by monotypy): Mesostoma testaceipes Cameron, 1905.
Marginopius Fahringer, 1935: 9. Type species (by monotypy): Opius (Marginopius) romani Fahringer, 1935.
Austroopius Szépligeti, 1900: 64. Type species (by monotypy): Austroopius novaguineensis Szépligeti, 1900 [examined].
Acidoxanthopius Fischer, 1972: 71 (as subgenus of Opius Wesmael, 1835). Type species (by original designation): Opius acidoxanthicidus Fullaway, 1949.

#### Diagnosis

(mainly after Wharton 2009). Hypopygium of ♀ enlarged, 0.3–0.5 times as long as length of metasoma, distinctly acute apically (Figs 13, 44, 65) and vein m-cu of fore wing 0.5–0.7 times vein 1-M (Figs 2, 14, 28, 55); pterostigma distinctly triangular (Figs 2, 55, 78, 90); scutellum slightly convex; second metasomal tergite strongly transverse, posterior width 4–7 times its median length (Fig. 5, but sometimes not separated from third tergite and nearby border only indicated by line of setae) and its anterior half usually without granulation, but sometimes distinct in *Psyttalia cyclogaster* (Fig. 17) and similar species; hypoclypeal depression wide and clypeus medium-sized (Fig. 19) or narrow (Figs 49, 71, 83, 95); precoxal sulcus impressed and usually crenulate medially; antenna of ♀ 1.1–1.7 times as long as fore wing; temple narrow (Figs 8, 32, 50, 96) or medium-sized (Figs 20, 84); vein m-cu of fore wing more or less antefurcal or interstitial (but more or less postfurcal in *Psyttalia cyclogaster* (Fig. 13) and similar species), gradually merging into vein 2-CU1 (Figs 28, 78) or angled with 2-CU1 (Figs 2, 13, 55, 90), straight or slightly (Fig. 2) to strongly curved; vein 1-CU1 of fore wing more or less widened (Figs 2, 28, 35, 66; but hardly so in *Psyttalia cyclogaster* (Fig. 13) and similar species); vein 2-SR+M of fore wing absent (Fig. 13) or present and more or less widened (Figs 2, 28, 55) or slender (Figs 55, 90); vein CU1b of fore wing present; second submarginal cell of fore rather elongate (Figs 2, 14); antero-medially pronotum at most with a transverse groove (Fig. 9) or with an shallow point-like pronope; mandible symmetrical, without extra protuberance (Fig. 86); medio-longitudinal carina of propodeum often present, but hardly so in *Psyttalia cyclogaster* (Fig. 17) and similar species); ovipositor sheath protruding far beyond apex of metasoma, its setose part usually 3–5 times as long as first metasomal tergite.

#### Biology.

Parasitoids of larvae of Tephritidae; mainly in fruits, but sometimes in buds, flowers or galls (Wharton 2009).

#### Distribution.

Cosmopolitan, except Nearctic and Neotropical regions. Wharton (2009) excluded *Psyttalia ovaliops* (Fischer, 1980) and *Psyttalia rufoflava* Fischer, 2001 (the only species known from the New World) because they belong to different New World species groups.

#### Notes.

Tobias and Jakimavičius (1986) synonymized *Phlebosema* Fischer, 1972 (as “*Phelbosema*”) with *Psyttalia*. This is not accepted here because the type species (*Opius discreparius* Fischer, 1963, from Japan) has a narrow elliptical pterostigma and the second metasomal tergite is granulate. Later Tobias included the type species in the subgenus Tolbia Cameron, 1907 (Tobias 1998). Both subgenera (*Phlebosema* and *Tolbia*) were synonymized with *Phaedrotoma* Foerster, 1863, by Li et al. (2013).

All known *Psyttalia* species from China have the setose part of ovipositor sheath about as long as the metasoma or slightly longer (= 3–5 times as long as first metasomal tergite). If the sheath is about twice as long as the metasoma, see the similar *Phaedrotoma daghestanicum* (Telenga, 1950) comb. n. that may occur in NW China. It is not included in *Psyttalia*, because the medio-posterior depression of the mesoscutum is present, vein CU1b of the fore wing is absent, the pterostigma is narrow, vein 1-CU1 of the fore wing is narrow, the precoxal sulcus is absent and the second metasomal tergite is as long as the third tergite (Fischer 1983). It is included in *Phaedrotoma* because it keys out there in the key by Li et al. (2013) and in the key below.

The genus *Psyttalia* Walker may be confused with *Psyttoma* van Achterberg & Li and some species of *Phaedrotoma* Foerster (Li et al. 2013), because of the acute hypopygium and far-protruding ovipositor. They can be separated as follows (for convenience *Rhogadopsis* is added because sometimes *Rhogadopsis* species are mistaken for *Psyttalia*).

**Table:** 

1	Scutellum distinctly protruding above level of mesoscutum; hypopygium of ♀ distinctly acute apically and about 0.3 times as long as metasoma **and** hind wing narrow; hind femur very robust, 2–3 times as long as wide; labrum slanted backwards, leaving a depression below clypeus; medio-anterior veins of hind wing of ♂ strongly widened	***Psyttoma* van Achterberg & Li, 2012**
–	Scutellum at level of mesoscutum; hypopygium of ♀ variable, **if** distinctly acute apically and about 0.3 times as long as metasoma **then** hind wing moderately wide and hind femur slender, 4–5 times as long as wide; labrum normal, without depression below clypeus; medio-anterior veins of hind wing of ♂ narrow	**2**
2	Hypopygium of ♀ often distinctly acute apically and 0.3–0.6 times as long as metasoma, **if** without narrow acute apex **then** vein 2-SR+M of fore wing distinctly widened medially; second metasomal tergite strongly transverse and shorter than third tergite; first discal cell of fore wing transverse (Fig. 28), but less so in *Psyttalia cyclogaster* (Fig. 14); vein m-cu of fore wing often gradually merging into vein 2-CU1 and more or less curved (Fig. 28); Old World	***Psyttalia* Walker, 1860**
–	Hypopygium of ♀ obtuse apically or nearly so and 0.1–0.3 times as long as metasoma; **if** rather acute apically and enlarged, **then** vein 2-SR+M of fore wing narrow medially, second tergite less transverse and about as long as third tergite; first discal cell of fore wing usually less transverse (Fig. 101); vein m-cu of fore wing usually angled with vein 2-CU1 and straight (Fig. 101); cosmopolitan	**3**
3	Propodeum with medio-longitudinal carina anteriorly; vein m-cu of fore wing often gradually merging into 2-CU1 and linear with vein 2-M or nearly so; vein 1r-m of hind wing less oblique and 0.6–1.0 times as long as vein 1-M (combined with a comparatively wide hind wing); anterior groove of metapleuron crenulate dorsally; vein CU1b of fore wing medium-sized	***Rhogadopsis* Brèthes, 1913**
–	Medio-longitudinal carina of propodeum absent anteriorly; vein m-cu of fore wing angled with vein 2-M, **if** rarely linear **then** angled with vein 2-CU1; vein 1r-m of hind wing usually distinctly oblique and 0.3–0.6 times as long as vein 1-M; at least dorsal half of anterior groove of metapleuron smooth; vein CU1b of fore wing usually short or absent, but sometimes medium-sized	***Phaedrotoma* Foerster, 1863**

#### Key to East Palaearctic and North Oriental species of the genus *Psyttalia* Walker

**Table:** 

1	Scutellum medio-posteriorly densely setose and micro-sculptured, and slightly protruding or pinched subposteriorly (Figs 16, 17); vein m-cu of fore wing distinctly postfurcal (Fig. 14) to subinterstitial; area behind stemmaticum with a small pit and in front of anterior ocellus with a smooth protuberance (Figs 20, 21; often absent or obsolescent in small specimens); propodeum largely finely rugose (Fig. 17); [hind femur 3.5–4.2 times as long as wide (Fig. 25); antenna with 26–39 segments; setose part of ovipositor sheath 0.43–0.57 times as long as fore wing and 1.31.8 times hind tibia; T2 more or less micro-sculptured; clypeus flattened, medium-sized trapezoid (Fig. 19)]	***Psyttalia cyclogaster* (Thomson, 1895)**
–	Scutellum medio-posteriorly with some setae and smooth, and flat subposteriorly (Figs 4, 37, 58, 68); vein m-cu of fore wing more or less antefurcal (Figs 2, 28, 55, 78, 90); area behind stemmaticum without a pit or pit minute and in front of anterior ocellus flat or with a narrow convex ridge (Figs 8, 32, 84, 96); propodeum at least partly smooth (Figs 5, 30, 64, 68, 93)	**2**
2	Propodeum with pair of complete, medium-sized and coarsely crenulate grooves sublaterally (Fig. 93); frons largely punctate-rugose in front of anterior ocellus (Fig. 96); vein SR of hind wing absent (Fig. 90); sixth tergite longer than fifth tergite or nearly as long and ivory (Figs 89, 99); vein m-cu of fore wing subparallel to vein 1-M, straight and vein 2-SR+M slender (Fig. 90); antenna with 52–53 segments	***Psyttalia spectabilis* van Achterberg, sp. n.**
–	Propodeum at most with pair of finely crenulate narrow grooves (Fig. 80) or with wide and incomplete crenulate grooves anteriorly (Figs 47, 64, 68); frons smooth in front of anterior ocellus, at most near antennal sockets sculptured (Figs 50, 72, 84); vein SR of hind wing indicated as faint depression (Fig. 78); sixth tergite shorter than fifth tergite or nearly as long and usually black or brownish yellow (Figs 12, 51, 73); vein m-cu of fore wing usually distinctly converging to vein 1-M posteriorly, more or less curved and vein 2-SR+M more or less widened (Figs 2, 28, 35, 55, 66, 78); antenna with 36–55 segments	**3**
3	Vein r of fore wing 0.7–1.0 times vein 2-SR (Fig. 28); vein 2-SR+M of fore wing distinctly widened (Fig. 28); antenna largely brownish yellow	**4**
–	Vein r of fore wing 0.3–0.5 times vein 2-SR (Figs 2, 35, 55, 66); vein 2-SR+M of fore wing hardly or not widened (Figs 2, 55, 78); antenna (except scapus and pedicellus) dark brown or brown	**6**
4	Vein 2-SR+M of fore wing 3.5–4.0 times as long as wide (Fig. 28); vein m-cu of fore wing weakly curved or straight (Fig. 28)	***Psyttalia incisi* (Silvestri, 1916)**
–	Vein 2-SR+M of fore wing about twice as long as wide; vein m-cu of fore wing strongly curved	**5**
5	Vein r of fore wing about 0.8 times vein 2-SR; vein 1-CU1 of fore wing about as long as vein cu-a	***Psyttalia makii* (Sonan, 1932)**
–	Vein r of fore wing about as long as vein 2-SR; vein 1-CU1 of fore wing at most 0.7 times as long as vein cu-a	***Psyttalia fletcheri* (Silvestri, 1916)**
6	Head directly narrowed behind eyes in dorsal view, eye 3–6 times longer than temple (Figs 8, 50); wing membrane subhyaline (Fig. 1); hypopygium of ♀ pale yellowish or pale brown medio-ventrally (Figs 12, 51); length of fore wing 2.8–3.4 mm; antenna of ♀ with 36–44 segments	**7**
–	Head gradually narrowed behind eyes in dorsal view, eye 1.8–2.5 times longer than temple (Figs 72, 84); wing membrane weakly to distinctly infuscate (Figs 66, 78); hypopygium of ♀ dark brown or brown medio-ventrally (Figs 73, 85); length of fore wing 4.5–5.5 mm; antenna of ♀ with 44–47 segments	**9**
7	Vein 1-CU1 of fore wing strongly widened and nearly as long as vein 2-CU1 (Figs 34–35); ocelli large (Fig. 40); frons smooth laterally; mesoscutum of ♂ with well-defined V-shaped pale yellow area (Fig. 37)	***Psyttalia latinervis* Wu & van Achterberg, sp. n.**
–	Vein 1-CU1 of fore wing at most moderately widened and much shorter than vein 2-CU1 (Figs 2, 55); ocelli smaller (Fig. 8); **if** rather large (Fig. 61) then frons punctate laterally (Fig. 61); mesoscutum of ♂ without distinct V-shaped area medio-posteriorly (Fig. 58), at most mesoscutum with rectangular yellowish brown area medially	**8**
8	OOL 2.0–2.4 times diameter of posterior ocellus and POL slightly longer than diameter of ocellus (Fig. 8); frons and vertex laterally largely smooth, except some punctulation (Fig. 8); medio-posterior triangular areola of propodeum short (Fig. 5); pterostigma dark brown medially (Fig. 2); vein 2-SR+M of fore wing about 0.4 times as long as vein m-cu (Fig. 2); base of hind tibia and hind tarsus brownish yellow (Fig. 12)	***Psyttalia carinata* (Thomson, 1895)**
–	OOL 1.2–1.7 times diameter of posterior ocellus and POL 0.8–1.0 times diameter of ocellus (Figs 50, 61); frons and vertex punctate laterally (Fig. 50); medio-posterior triangular areola of propodeum variable, often longer (Figs 48, 63, 64); pterostigma pale brown medially (Figs 44, 54, 55); vein 2-SR+M of fore wing 0.6–0.8 times as long as vein m-cu (Figs 45, 54, 55); base of hind tibia often and hind tarsus largely dark brown (Fig. 57)	***Psyttalia majocellata* Wu & van Achterberg, sp. n.**
9	Mesosoma orange brown, contrasting with mainly black metasoma (Fig. 65); hind femur robust and 2.9–3.3 times as long as wide (Fig. 73); fore wing distinctly infuscate (Fig. 66); vein 2-SR+M of fore wing rather widened (Fig. 66); legs yellowish brown (Fig. 65); vein 3-SR of fore wing 1.4–1.8 times as long as vein 2-SR (Fig. 66)	***Psyttalia romani* (Fahringer, 1935)**
–	Mesosoma mainly black or dark brown as metasoma (Fig. 77); hind femur slenderer and 3.5–3.9 times as long as wide (Fig. 85); fore wing slightly infuscate (Fig. 78); vein 2-SR+M of fore wing slightly widened (Fig. 78); legs brownish yellow (Fig. 77); vein 3-SR of fore wing 1.4–1.5 times as long as vein 2-SR (Fig. 78)	***Psyttalia sakhalinica* (Tobias, 1998)**

**Figure 1.**
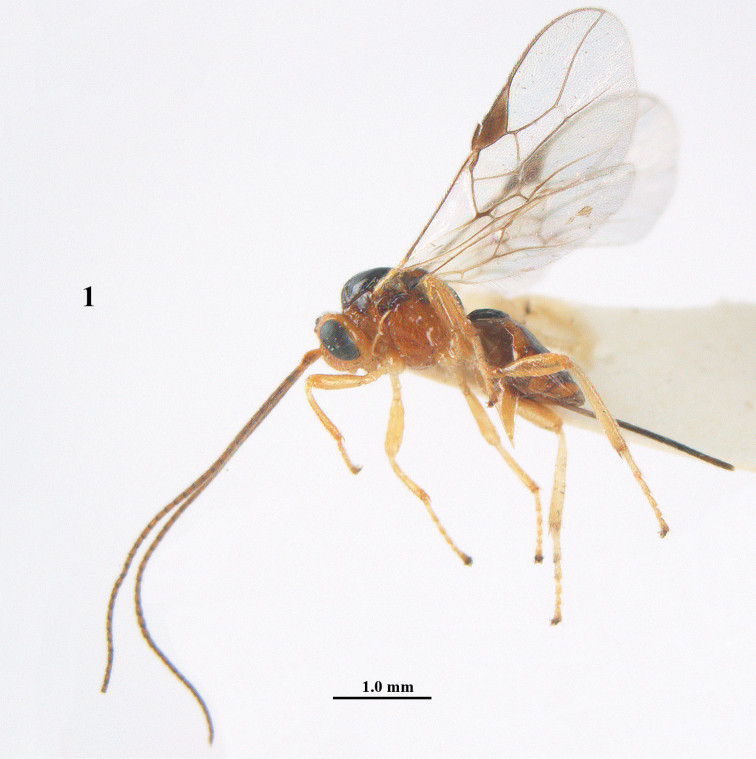
*Psyttalia carinata* (Thomson), ♀, holotype of *Opius brevitemporalis* Tobias, habitus lateral.

**Figures 2–12.**
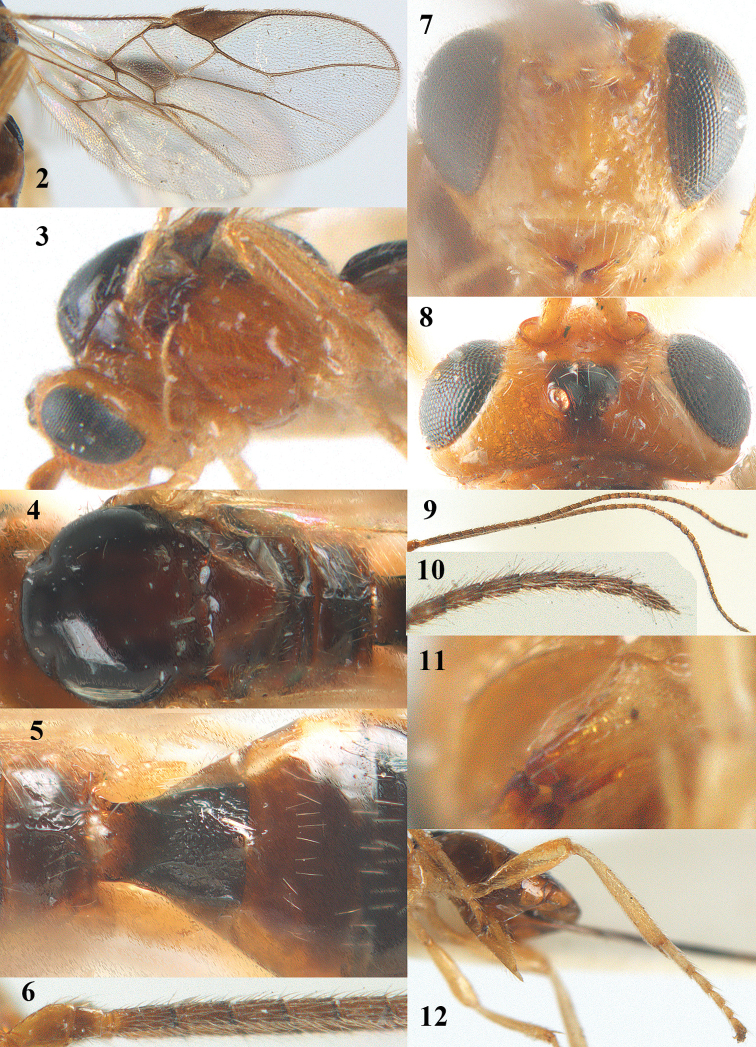
*Psyttalia carinata* (Thomson), ♀, holotype of *Opius brevitemporalis* Tobias. **2** wings **3** head and mesosoma lateral **4** mesosoma dorsal **5** propodeum and first–third metasomal tergites dorsal **6** base of antenna **7** head anterior **8** head dorsal **9** antenna **10** apex of antenna **11** mandible lateral **12** hind leg and hypopygium lateral.

**Figure 13.**
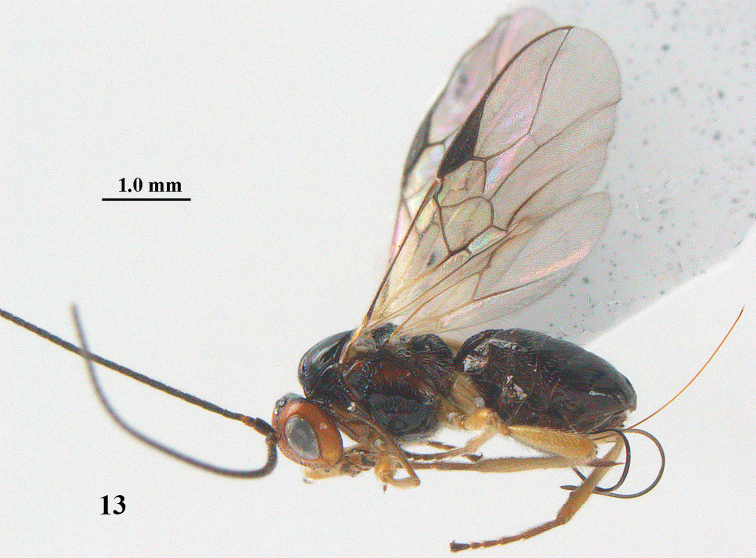
*Psyttalia cyclogaster* (Thomson), ♀, China, Ningshan, habitus lateral.

**Figures 14–24.**
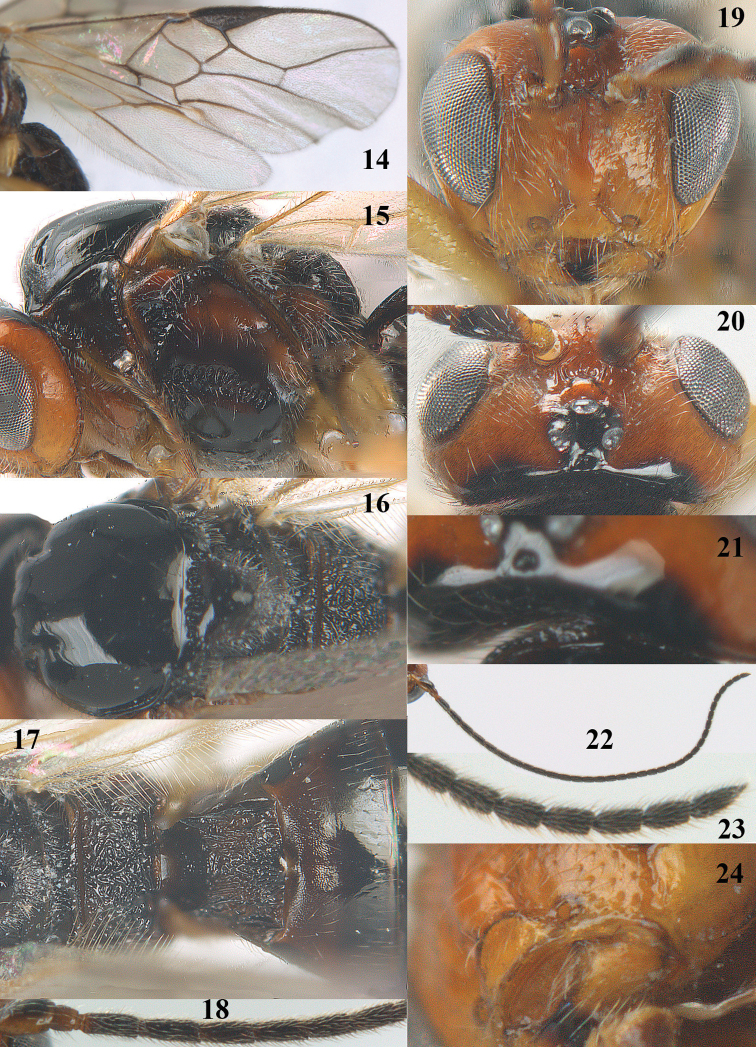
*Psyttalia cyclogaster* (Thomson), ♀, China, Ningshan. **14** wings **15** mesosoma lateral **16** mesosoma dorsal **17** propodeum and first–third metasomal tergites dorsal **18** base of antenna **19** head anterior **20** head dorsal **21** detail of posterior part of head and pronotum dorsal **22** antenna **23** apex of antenna **24** mandible antero-lateral.

**Figures 25–27.**
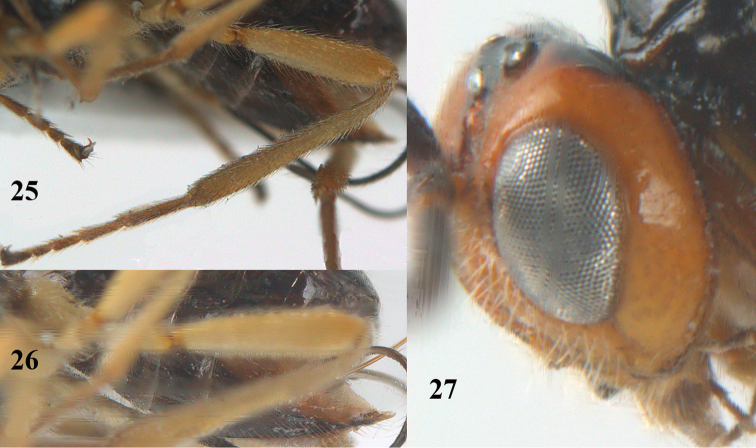
*Psyttalia cyclogaster* (Thomson), ♀, China, Ningshan. **25** hind leg lateral **26** hypopygium lateral **27** head lateral.

**Figures 28–32.**
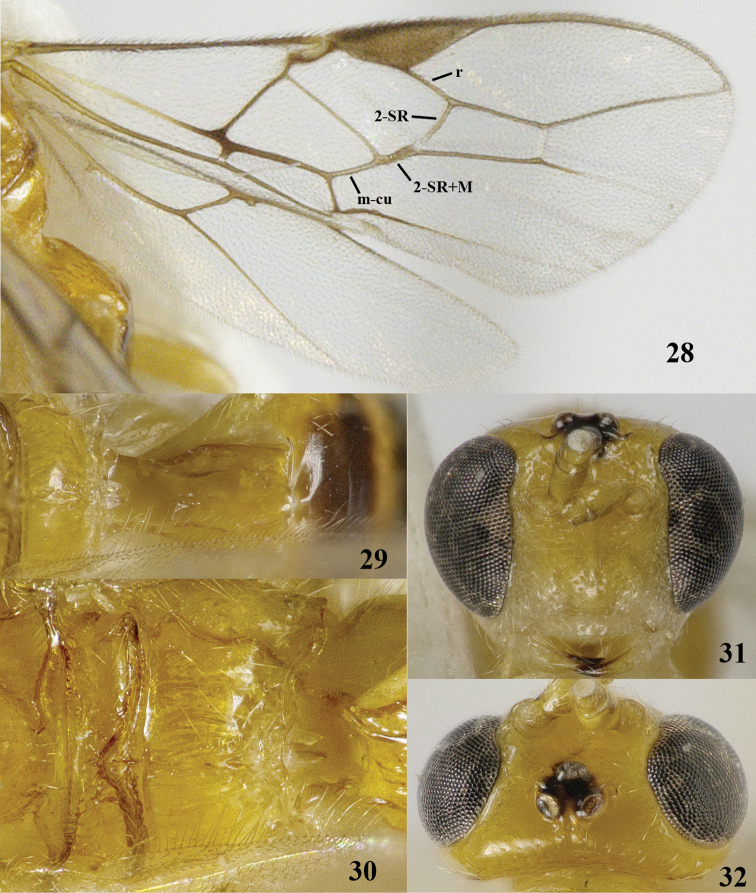
*Psyttalia incisi* (Silvestri), ♂, China, Fujian. **28** wings **29** first metasomal tergite dorsal **30** propodeum dorsal **31** head anterior **32** head dorsal.

**Figure 33.**
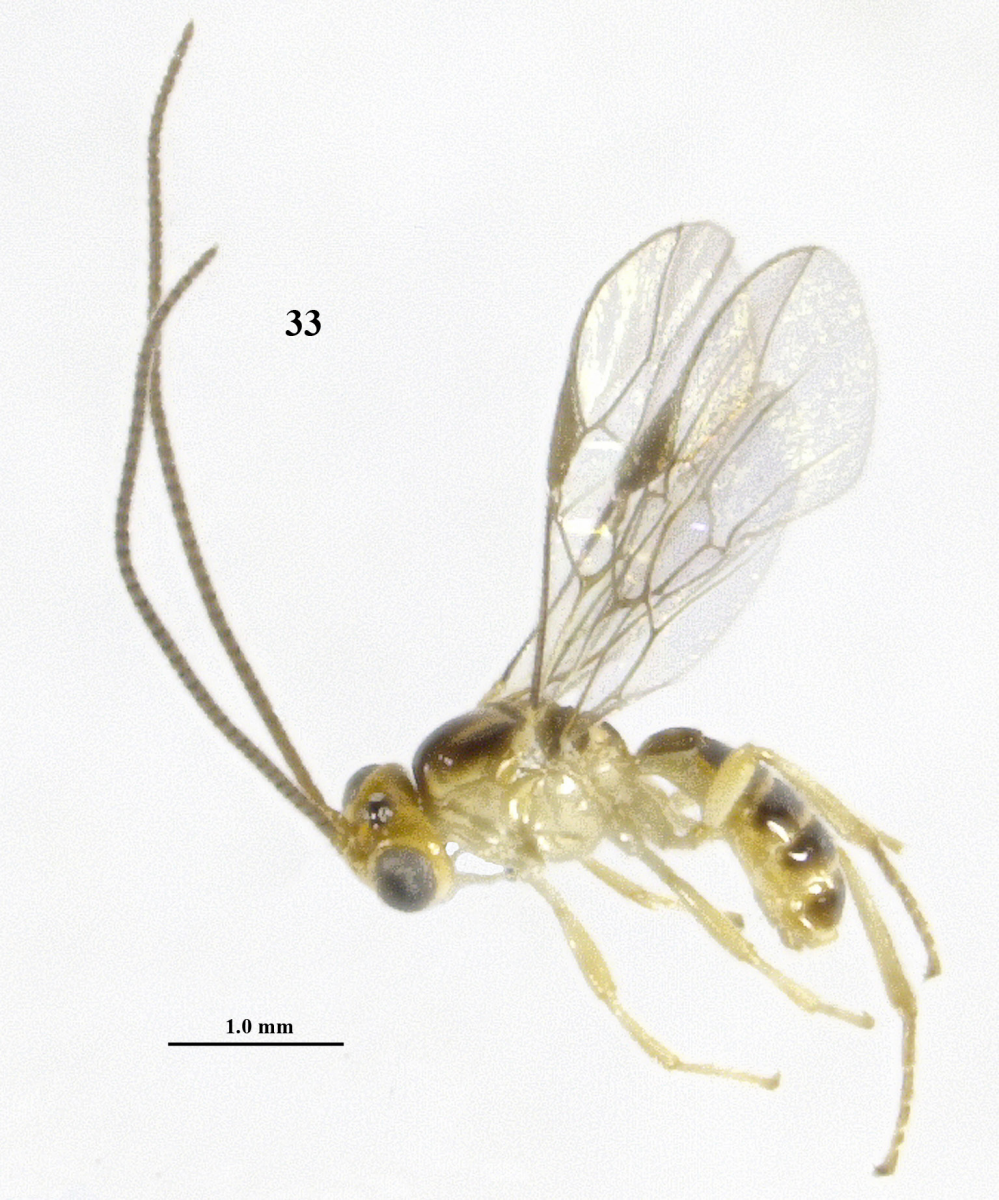
*Psyttalia latinervis* sp. n., ♂, holotype, habitus lateral.

**Figures 34–43.**
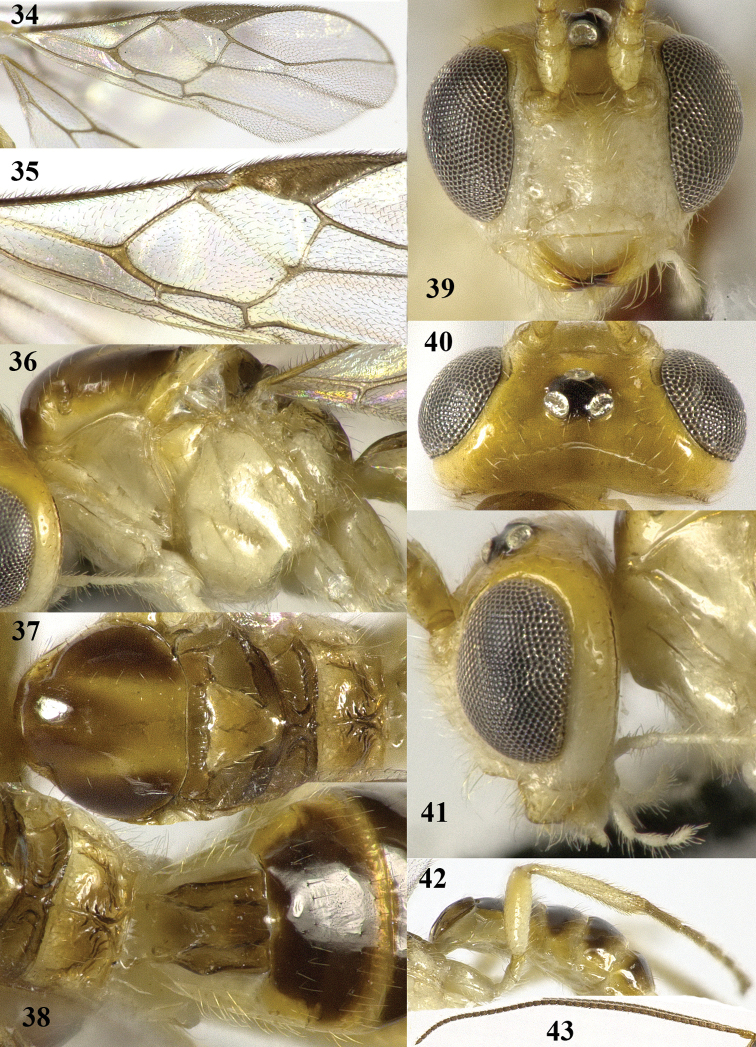
*Psyttalia latinervis* sp. n., ♂, holotype. **34** wings **35** detail of middle third of fore wing **36** mesosoma lateral **37** mesosoma dorsal **38** propodeum and first–third metasomal tergites dorsal **39** head anterior **40** head dorsal **41** head lateral **42** hind leg **43** antenna.

**Figure 44.**
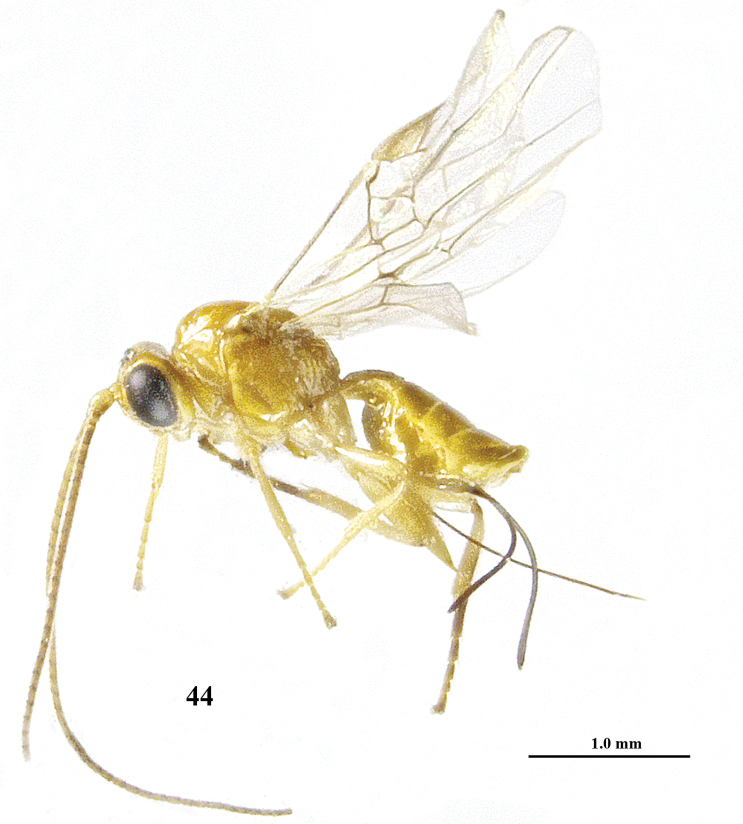
*Psyttalia majocellata* sp. n., ♀, holotype, habitus lateral.

**Figures 45–52.**
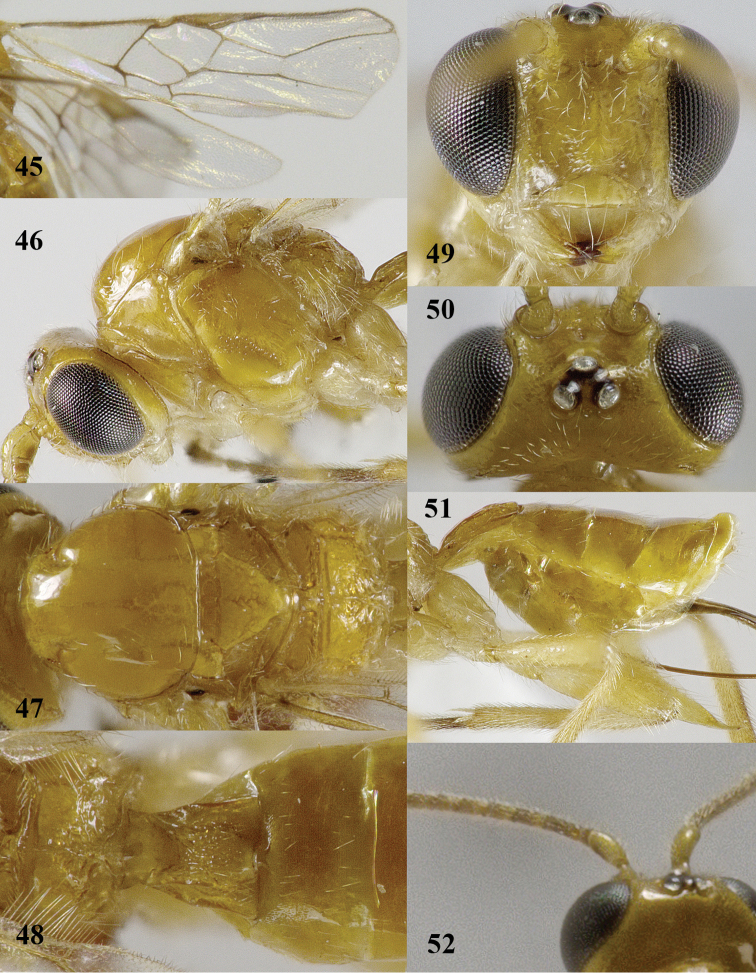
*Psyttalia majocellata* sp. n., ♀, holotype. **45** wings **46** head and mesosoma lateral **47** mesosoma dorsal **48** propodeum and first–third metasomal tergites dorsal **49** head anterior **50** head dorsal **51** hind femur and hypopygium lateral **52** base of antenna.

**Figure 53.**
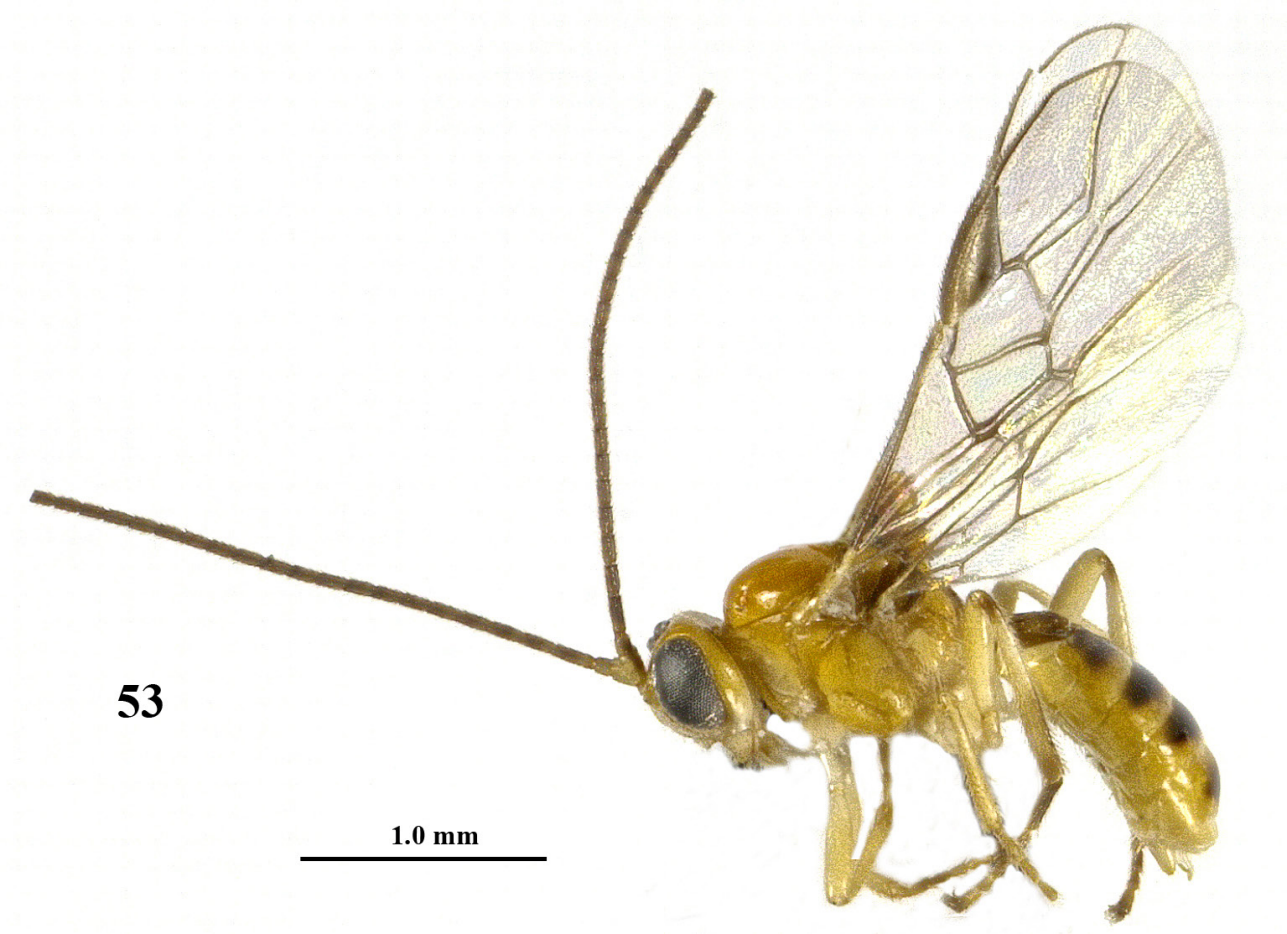
*Psyttalia majocellata* sp. n., ♂ paratype, habitus lateral.

**Figures 54–64.**
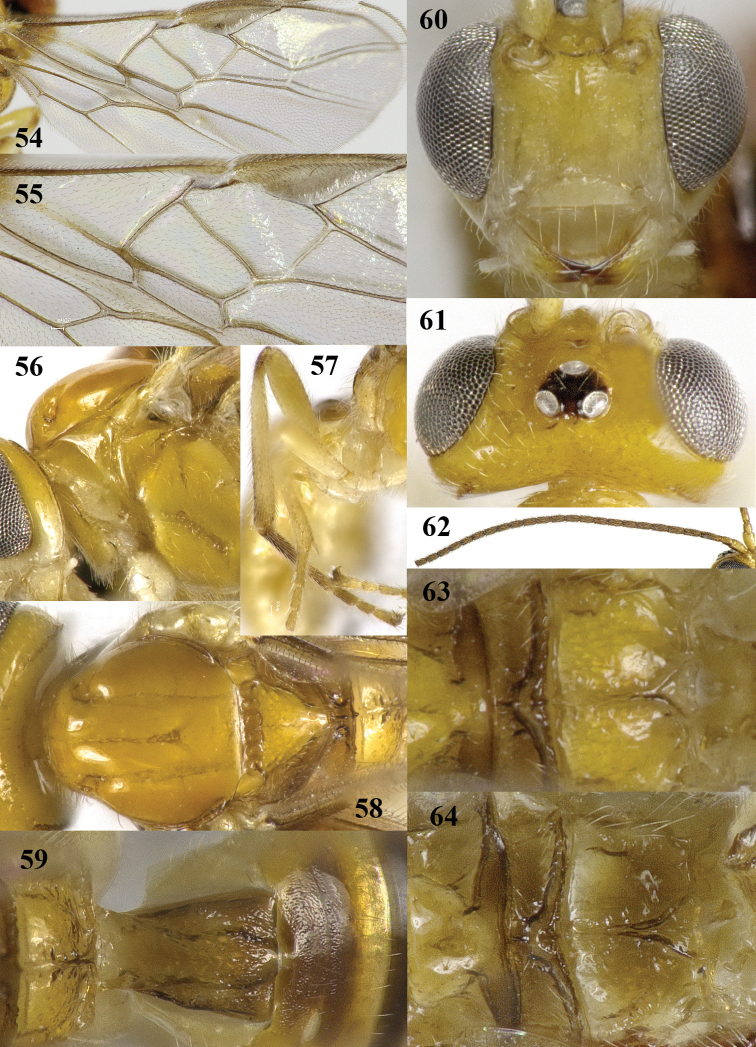
*Psyttalia majocellata* sp. n., ♂ paratype, but 64 of ♀ holotype. **54** wings **55** detail of middle third of fore wing **56** mesosoma lateral **57** hind leg **58** mesosoma dorsal **59** propodeum and first–third metasomal tergites dorsal **60** head anterior **61** head dorsal **62** antenna **63–64** metanotum and propodeum dorsal.

**Figure 65.**
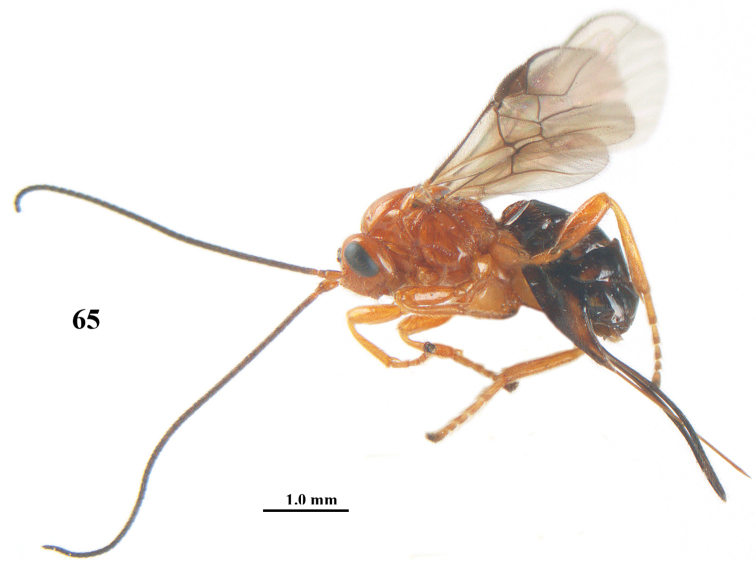
*Psyttalia romani* (Fahringer), ♀, Russia, Novorossijka, habitus lateral.

**Figures 66–76.**
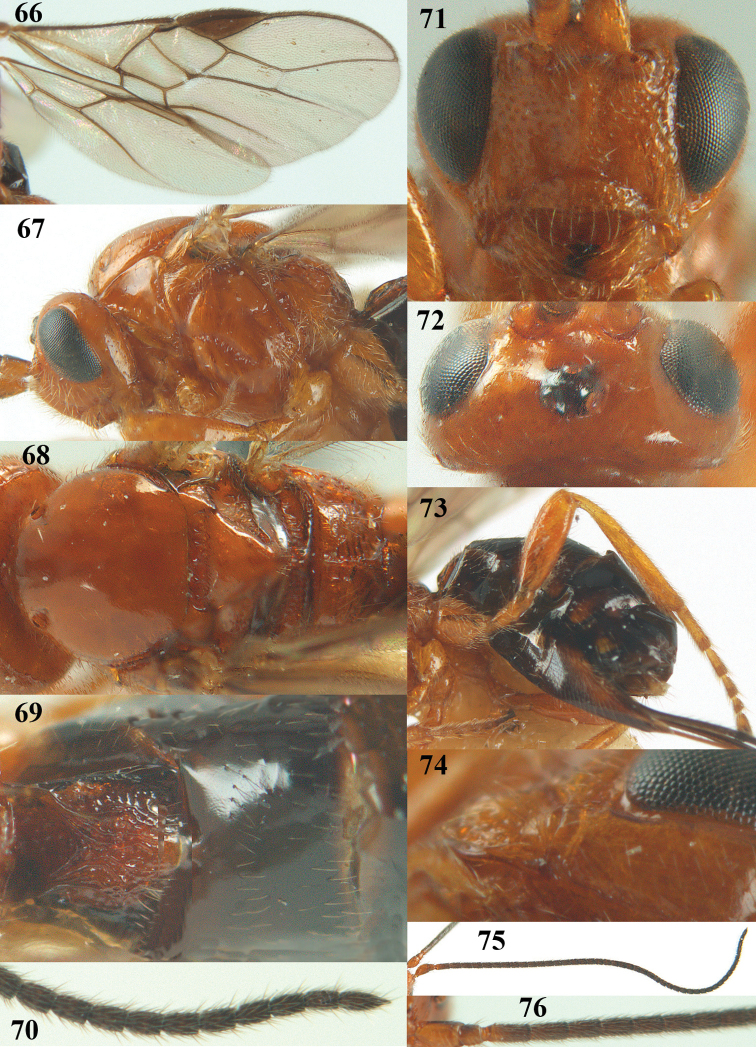
*Psyttalia romani* (Fahringer), ♀, Russia, Novorossijka. **66** wings **67** head and mesosoma lateral **68** mesosoma dorsal **69** first–third metasomal tergites dorsal **70** apex of antenna **71** head anterior **72** head dorsal **73** hind leg and hypopygium lateral **74** mandible lateral **75** antenna **76** base of antenna.

**Figure 77.**
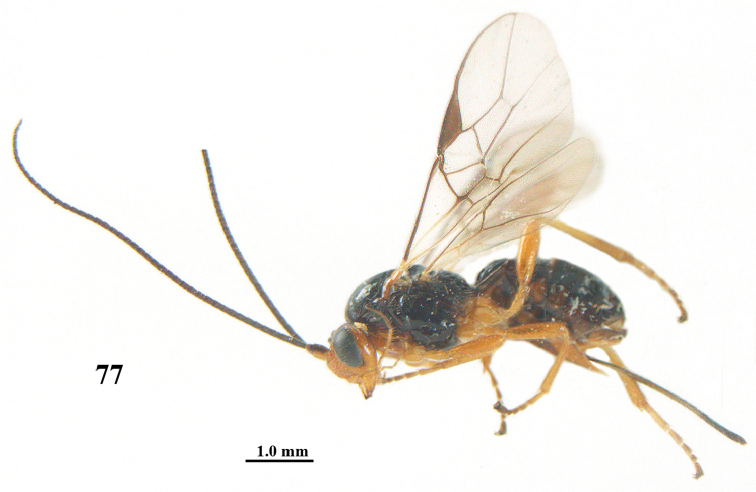
*Psyttalia sakhalinica* (Tobias), ♀, holotype, habitus lateral.

**Figures 78–88.**
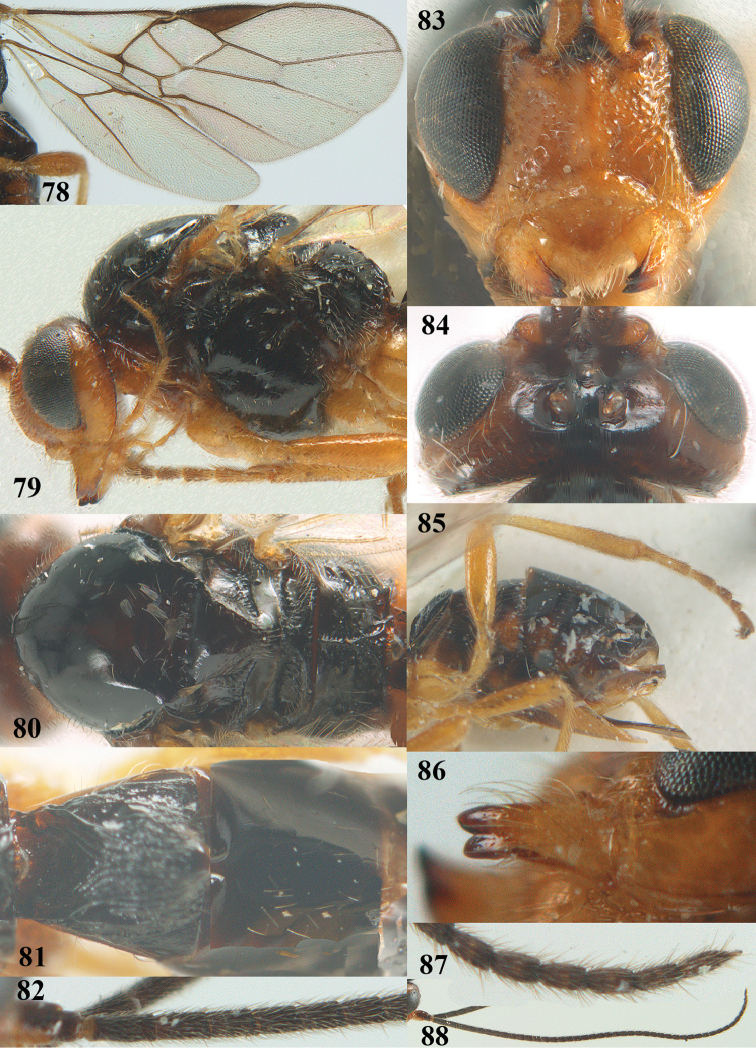
*Psyttalia sakhalinica* (Tobias), ♀, holotype. **78** wings **79** head and mesosoma lateral **80** mesosoma dorsal **81** first–third metasomal tergites dorsal **82** base of antenna **83** head anterior **84** head dorsal **85** hind leg and hypopygium lateral **86** mandible lateral **87** apex of antenna **88** antenna.

**Figure 89.**
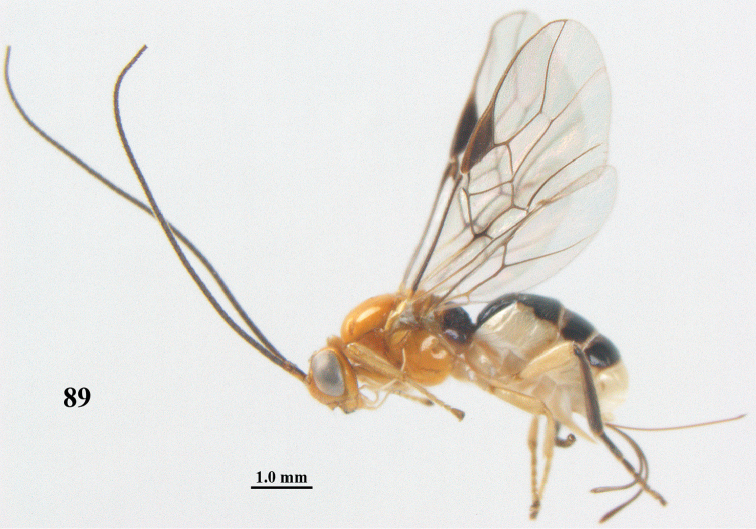
*Psyttalia spectabilis* sp. n., ♀, holotype, habitus lateral.

**Figures 90–99.**
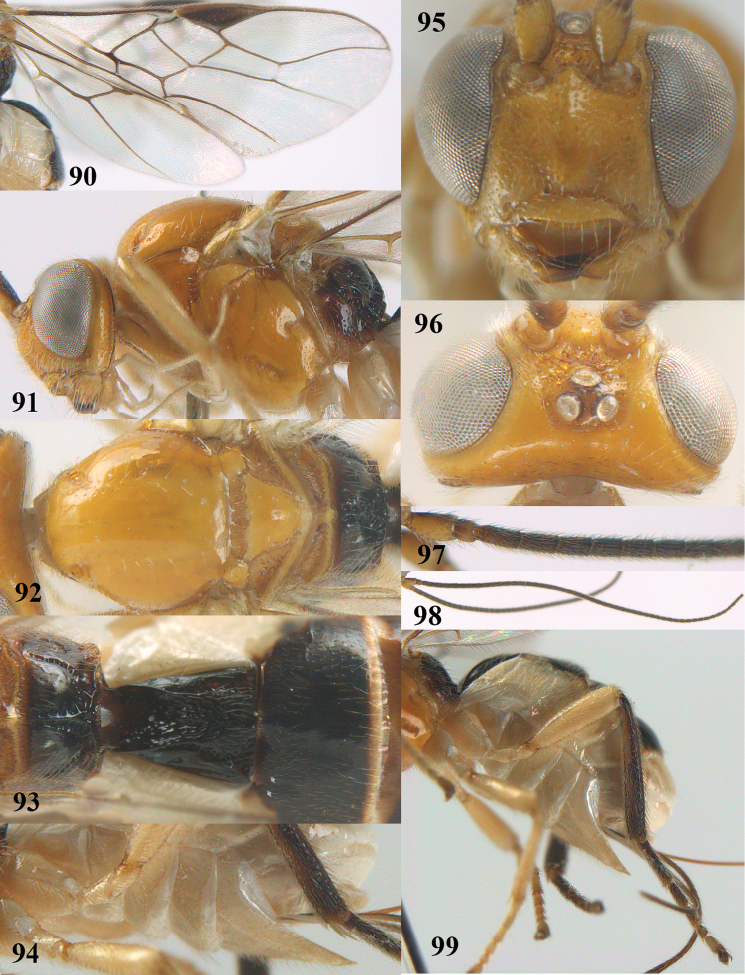
*Psyttalia spectabilis* sp. n., ♀, holotype. **90** wings **91** head and mesosoma lateral **92** mesosoma dorsal **93** propodeum and first–third metasomal tergites dorsal **94** hypopygium lateral **95** head anterior **96** head dorsal **97** base of antenna **98** antenna **99** hind leg and hypopygium lateral.

**Figure 100.**
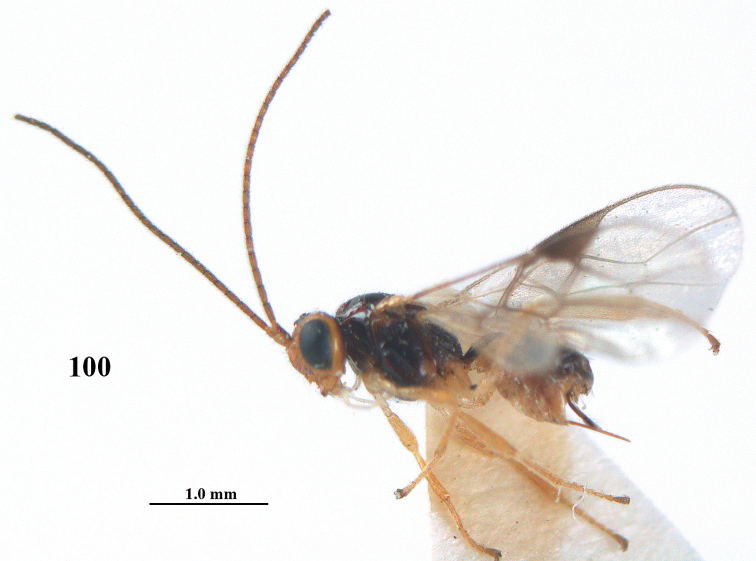
*Rhogadopsis mediocarinata* (Fischer), ♀, holotype of *Opius vacuus* Tobias, habitus lateral.

**Figures 101–110.**
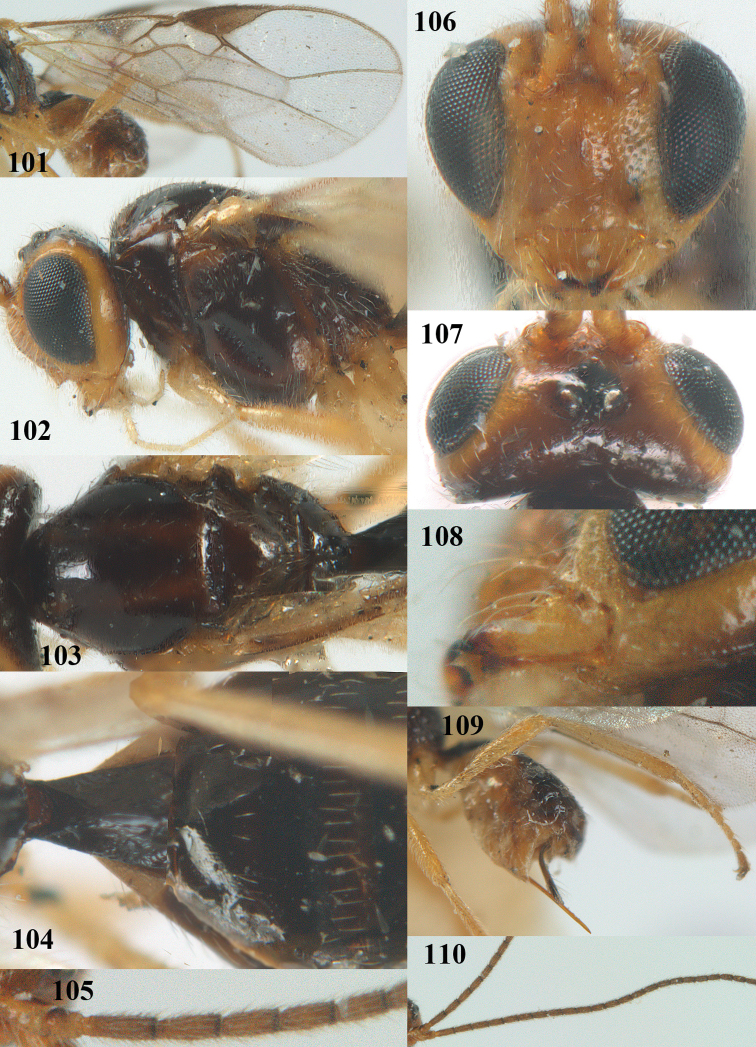
*Rhogadopsis mediocarinata* (Fischer), ♀, holotype of *Opius vacuus* Tobias. **101** wings **102** head and mesosoma lateral **103** mesosoma dorsal **104** first–third metasomal tergites dorsal **105** base of antenna **106** head anterior **107** head dorsal **108** mandible lateral **109** hind leg and hypopygium lateral **110** antenna.

### Psyttalia carinata

Taxon classificationAnimaliaHymenopteraBraconidae

(Thomson, 1895) s.l.

Figs 1 , 2–12

Opius carinatus Thomson, 1895: 2177.
Opius (Psyttalia) carinatus : Fischer 1972: 335–337; Tobias 1998: 613.
Psyttalia carinata : Fischer and Koponen 1999: 144; Belokobylskij et al. 2003: 396; van Achterberg 2004c: FE on-line database.
Opius rhagoleticola Sachtleben, 1934: 76; Fischer 1972: 344–346; Belokobylskij et al. 2003: 396 (as synonym of Psyttalia carinata).
Psyttalia rhagoleticola : Fischer and Koponen 1999: 144; Tobias 2000: 12.
Opius (Psyttalia) ophthalmicus Tobias, 1977: 425, 430, 1998: 613; Fischer 1984: 114–117. **Syn. n.** (examined).
Psyttalia ophthalmica : Wharton 1997: 23; Tobias 2000: 12.
Opius (Psyttalia) brevitemporalis Tobias, 1998: 613. **Syn. n.** (examined).
Psyttalia brevitemporalis : Tobias 2000: 12.

#### Type material.

Lectotype of *Opius carinatus* here designated, ♀ (ZIL), “Broa” [= North Gottland, **Sweden**], 12–12.vii.[18]50”; 1 paralectotype, ♂ (ZIL) with same label data as lectotype; 1 paralectotype, ♂ (ZIL), “Gott”, “*carinatus* m. “, “*Opius carinatus* Th.”. Paratypes of *Opius rhagoleticola*: 3 ♀ (RMNH, ZJUH), “Cotypus”, “[**Germany**], Naumburg, 1932, aus *Rhagoletis cerasi*, Thiem”, “*Opius rhagoleticolus* Sachtl.” Holotype of *Opius ophthalmicus* ♀ (ZISP), “[**Russia**:], Primorskij kraj, okr. Ussurskiska, 13.ix.[1]968, Kandybina”, “*Rhagoletis alternatum* Flln., Kandybina det.”, “Litsinka v plodach zhipovnika *Rosa*”, “Holotypus *Opius ophthalmicus* Tobias”; 1 paratype, ♀ (ZISP), same data as holotype. Holotype of *Opius brevitemporalis*, ♀ (ZISP), “[Russia:], Primorskij kraj, Spassk, 21.viii.1987, G. Belokobylskaja”, “*Opius brevitemporalis* sp. n., det. Tobias ‘95”, “Holotypus *Opius brevitemporalis* Tobias”; 1 paratype, ♀ (ZISP), “Primorskij kraj, zap. Kedrovaja Pad, 25.ix.[1]968, Kandybina”, “[ex] *Myioleja sinensis* Zia, Kandybina det.”, “[ex] *Chaetostoma continuans* Zia & Chen”, “Litsinka v plodach shimolosti *Lonicera maackii* Rupr.”; “Paratypus *Opius brevitemporalis* Tobias”.

#### Additional material.

1 ♂ (ZISP), “[Russia], Ilmenskij zapoved, Tseljainskoj obl., 15.vii.[1]959, Tobias” (det. Tobias as *Opius carinatus*); 3 ♀ (ZISP), id., but 18.vii.1958. Additional specimens (ZISP) of *Psyttalia carinata* with complete yellowish mesoscutum examined from Gravan, Bijsp, Altajskij kraj, Karagand. Obl., Toshska Obl. (Russia) and Kizhinev (Moldova).

#### Comparative diagnosis.

*Psyttalia carinata* is a widespread Palaearctic species with the head distinctly narrower behind the eyes in dorsal view (eye 2.5–5 times longer than temple) and medium-sized ocelli (Fig. 8). This species is very similar to SW. Palaearctic and Afrotropical *Psyttalia concolor* (Szépligeti, 1910) as indicated by Fischer (1972); *Psyttalia carinata* differs by having mesosoma dorsally and the first metasomal tergite mainly or entirely black or dark brown (*vs* brownish or reddish yellow in *Psyttalia concolor*), vein cu-a of fore wing about as long as vein 1-CU1 (*vs* vein cu-a shorter than 1-CU1) and temple slightly less distinctly narrowed behind eyes (*vs* more directly narrowed) and by largely different spectrum of hosts belonging to *Carpomya*, *Chetostoma*, *Myoleja* and *Rhagoletis* species (*vs* *Anastrepha*, *Bactrocera*, *Capparimyia*, *Carpomya*, *Ceratitis*, *Dacus*, *Euphranta*, *Rhagoletis* and *Synclera* spp.).

#### Description.

Holotype of *Opius brevitemporalis*, ♀, length of body 2.8 mm, of fore wing 3.3 mm.

*Head*. Antenna with 40 segments, bristly and erect setose and 1.5 times as long as fore wing; third segment 1.2 times as long as fourth segment, length of third, fourth and penultimate segments 2.6, 2.2 and 2.3 times their width, respectively (Figs 6, 10); length of maxillary palp 0.9 times height of head; length of eye in dorsal view 4.2 times temple (Fig. 8); temple in dorsal view shiny, smooth and with sparse setae; OOL: diameter of ocellus: POL = 10:5:6; area behind stemmaticum reclivous and with minute pit (Fig. 8); face coarsely punctate with interspaces wider than diameter of punctures, shiny, with a smooth medio-longitudinal convexity widened ventrally (Fig. 7); frons slightly depressed behind antennal sockets and with some oblique striae; in front of anterior ocellus with slightly convex ridge, shiny, smooth and glabrous but laterally setose and punctulate (Fig. 8); labrum slightly depressed; clypeus transverse, sparsely punctate, convex, and its ventral margin truncate and narrow (Fig. 7); width of clypeus 4.3 times its maximum height and 0.7 times width of face; hypoclypeal depression wide and deep (Figs 7, 11); malar suture wide and shallow, punctate between malar suture and clypeus; mandible not twisted, apically moderately narrowed and with both teeth wide; mandible normal basally and with narrow ventral carina (Fig. 11); occipital carina remains far removed from hypostomal carina and dorsally largely absent; hypostomal carina narrow ventrally.

*Mesosoma*. Length of mesosoma 1.2 times its height; dorsal pronope minute, round; pronotal side largely smooth, but posterior groove dorsally crenulate (Fig. 3); propleuron slightly convex; epicnemial area smooth dorsally; precoxal sulcus medially medium-sized and only medially distinctly crenulate, absent anteriorly and posteriorly (Fig. 3); remainder of mesopleuron smooth and shiny; pleural sulcus smooth ventrally; mesosternal sulcus moderately deep, narrow and finely crenulate; postpectal carina absent; mesoscutum very shiny and glabrous (Fig. 4); notauli only anteriorly as pair of finely crenulate impressions and absent on disc; scutellar sulcus deep and with 6 short crenulae, parallel-sided medially; scutellum moderately convex and smooth, but apically sparsely punctate and setose (Fig. 4); metanotum with a protruding medio-longitudinal carina anteriorly and very finely crenulate posteriorly; surface of propodeum smooth and shiny except for rugose area near distinct and reversed Y-shaped median carina (Fig. 5), lateral grooves shallow and sparsely crenulate or smooth and anterior groove parallel-sided medially (Fig. 5).

*Wings*. Fore wing: 1-SR distinctly longer than wide and linear with 1-M (Fig. 2); pterostigma wide triangular (Fig. 2); 1-R1 ending at wing apex and 1.6 times as long as pterostigma (Fig. 2); r linear with 3-SR and medium-sized; r-m not tubular; r:3-SR:SR1 = 5:33:73; 2-SR:3-SR:r-m = 22:33:11; 1-M straight and SR1 curved; m-cu distinctly antefurcal and slightly curved, 2-M+CU1 moderately widened (as apex of M+CU1: Fig. 2) and 0.4 times as long as m-cu; cu-a distinctly postfurcal and 1-CU1 widened; 1-CU1:2-CU1= 5:23; first subdiscal cell closed; CU1b medium-sized; only apex of M+CU1 sclerotized. Hind wing: 1-M of hind wing straight, resulting in subparallel-sided cell apically; M+CU:1-M:1r-m = 5:5:4; cu-a straight; m-cu absent; SR slightly indicated apically.

*Legs*. Length of femur, tibia and basitarsus of hind leg 3.4, 8.0 and 4.4 times as long as width, respectively (Fig. 12); hind femur with rather long setae, tarsus and tibia densely setose.

*Metasoma*. Length of first tergite 1.2 times to its apical width, convex medio-posteriorly, its surface strongly and irregularly rugose-punctate (Fig. 5), dorsal carinae strong in its basal half and area below depressed; second suture slightly indicated; basal depressions of second tergite large and tergite 0.9 times as long as third tergite; second and following tergites smooth, shiny and sparsely setose; combined length of second and third metasomal tergites 0.25 times total length of metasoma; length of setose part of ovipositor sheath 0.52 times fore wing, 3.8 times first tergite, 2.4 times hind femur, 1.6 times hind tibia and 1.2 times metasoma; hypopygium about 0.5 times as long as metasoma, distinctly acute apically and about reaching apex of metasoma (Fig. 12).

*Colour*. Brownish yellow, but stemmaticum and area behind it, mesoscutum, metanotum, propodeum, first tergite and ovipositor sheath mainly black or blackish brown; antenna (except scapus and apically pedicellus), scutellum, pronotum and mesopleuron dorsally, second third tergites medially, fourth and fifth tergites (except lateral patch), sixth tergite medially, pterostigma and veins dark brown; remainder of sixth tergite yellowish; palpi, mandible (but teeth dark brown), tegulae and legs pale yellow; fore wing membrane subhyaline.

*Male*. Except for the sexual differences males are (as in other spp.) very similar to females; in general the size is less and more often than in females the metasomal tergites are darkened.

*Variation*. Length of fore wing 2.9–3.3 mm; antenna of ♀ with 35(1), 38(1), 39(1) and 40(1) segments, of ♂ 39(1); first tergite 1.1–1.2 times as long as its apical width; hind femur 3.4–4.2 times as long as wide; setose part of ovipositor sheath 0.50–0.54 times as long as fore wing, 0.8–1.1 times mesosoma and 1.5–1.7 times hind tibia; middle of mesoscutum black, chestnut brown or brown; area behind stemmaticum and scutellum dark brown to brownish yellow.

*Variation of type series*. The holotype of *Psyttalia ophthalmica* differs from typical *Psyttalia carinata* by having body partly dark brown and remainder yellowish brown, and scutellum with some setae and punctures posteriorly. These punctures are sometimes also present in typical *Psyttalia carinata* and both have been reared from *Rhagoletis alternata* (Fallén) (rose hip fly; Tephritidae). *Psyttalia brevitemporalis* has a similar scutellum (Fig. 4), but has the body largely dark brown dorsally and the holotype has the eye in dorsal view 4.2 (paratype 5.2) times as long as temple (4.2 times in holotype of *Psyttalia ophthalmica*, up to 3.8 times in *Psyttalia carinata*). According to Tobias (1998) *Psyttalia carinata* has the upper half of the mesopleuron granulate and *Psyttalia rhagoleticola* has it completely smooth, but clean specimens have always the mesopleuron smooth and shiny dorsally. The length of the temple in dorsal view seems to be variable. The W. Palaearctic specimens have the eye in dorsal view 2.5 times as long as temple (see fig. 267 in Fischer 1972) up to 3.8 times. In the East Palaearctic *Psyttalia brevitemporalis* and *Psyttalia ophthalmica* it varies between 4.2–5.2 times and because we could not find additional differences (except some variation in colour), we assume the variation is clinal. Therefore, we treat *Psyttalia carinata* *sensu lato* in this paper and synonymize both species under *Psyttalia carinata*.

#### Distribution.

Armenia; Austria; Bulgaria; Czech Republic; Finland; France; Germany; Hungary; Italy; Kazakhstan; Kyrgyzstan; Lithuania; Moldova; Netherlands (new record); Norway (id.); Poland; Russia (including Far East); Sweden; Switzerland; Uzbekistan and former Yugoslavia; introduced into Canada.

#### Biology.

Endoparasitoid of *Rhagoletis*, *Myoleja*, *Chetostoma* and *Carpomya* species (Tephritidae) in fruits.

#### Notes.

In ZJUH there is a similar female from S. China (Yunnan, Simao, 1982, Shiqing Yang, No. 826893) which most likely represents another new species. It has similar small ocelli and smooth frons, but the entirely mesoscutum is yellow, the base of the hind tibia is dark brown, the head is less transverse and vein m-cu of the fore wing is slightly longer than 2-SR+M (as in *Psyttalia majocellata* sp. n.). Differs from *Psyttalia majocellata* sp. n. by the largely dark brown second–fifth tergites of ♀ (*vs* yellow in ♀ of *Psyttalia majocellata*), the smaller ocelli, the dark brown middle of the pterostigma of ♀ and the less sculptured frons.

### Psyttalia cyclogaster

Taxon classificationAnimaliaHymenopteraBraconidae

(Thomson, 1895) comb. n.

Figs 13 , 14–24 , 25–27

Opius (Opius) cyclogaster Thomson, 1895: 2178 (examined).
Opius (Psyttalia) cyclogaster : Fischer 1972: 340–341.
Coeloreuteus formosanus Watanabe, 1934: 188; Chou 1981: 74; Chen and He 1997: 108. **Syn. n.**
Opius (Lissosema) proclivis Papp, 1981: 155–157. **Syn. n.** (examined).
Opius (Psyttalia) subcyclogaster Tobias, 1998: 612. **Syn. n.** (examined).
Psyttalia subcyclogaster : Tobias 2000: 12.
Opius (Psyttalia) darasunicus Tobias, 1998: 612. S**yn. n.** (examined).
Psyttalia darasunica : Tobias 2000: 12.
Opius (Psyttalia) cyclogastroides Tobias, 1998: 613. **Syn. n.** (examined).
Psyttalia cyclogastroides : Tobias 2000: 12.
Psyttalia extensa Weng & Chen, 2001: 84–86; Chen and Weng 2005: 150–151. **Syn. n.**
Rhogadopsis longicaudifera Li & van Achterberg, 2013: 151–154. **Syn. n.**

#### Type material.

Lectotype of *Opius cyclogaster* here designated, ♀ (ZIL), “[**France**:] Delazy, [1872]”, “*cyclogaster* m., “O. *cyclogaster* Th.”. Holotype of *Opius proclivis*, ♀ (TMAB), “**Korea**, prov. South Pyongan, Za-mo san, 60 km NE from Pyongyan, 2.ix.1971”, “No. 231, leg. S. Horvatovich et J. Papp”, “Holotypus ♀ % Opius (Lissosema) proclivis sp. n., Papp J., 1981”, “Hym. Typ. No. 2841, Museum Budapest”, “*Rhogadopsis* ♀ *proclivis* Papp, det. Papp J., 2012”. Holotype of *Opius subcyclogaster*, ♀ (ZISP), “[**Russia**:], Zabajkalsk, Tsitin., step, 1.vii.[1]975, Kasparjan”, “*Opius subcyclogaster* sp. n., Tobias det. 1998”, “Holotypus *Opius subcyclogaster* Tobias”. Holotype of *Opius darasunicus*, ♀ (ZISP), “[Russia:], 9 km S Kurorta, Darasun, Tsit. Obl., 27.vi.[1]975, Kasparjan”, “*Opius darasunicus* sp. n., Tobias det. 1998”, “Holotypus *Opius darasunicus* Tobias”. Holotype of *Opius cyclogastroides*, ♀ (ZISP), “[Russia:], Primorskij kraj, 20 km YuV Ussurijska, na svet, 18-21.vii.1996, S. Belokobylskij”, “*Opius cyclogastroides* sp. n., Tobias det. 1998”, “Holotypus *Opius cyclogastroides* Tobias”; 1 paratype, ♀ (ZISP), “[Russia:], Primorskij kraj, 10 km YuYuZ Partizanska, les, opushki, 12–13.vii.1996, S. Belokobylskij”, “Paratypus *Opius cyclogastroides* Tobias”. Holotype of *Rhogadopsis longicaudifera*, ♀ (ZJUH), “S. **China**: **Hunan**, Yongzhou, Jiangyong, Yuankou, 28.v.1988, Jian-Ping Liu, No. 181”.

#### Additional material.

1 ♀ (ZISP), “[**Japan**: Kyushu], Miyazaki, Yatake, 700 m, Shiiba-mura, 21.vii.1992, V. Makarkin”; 1 ♀ (ZISP), “[**Russia**:], 9 km S Kurorta, Darasun, Tsit. Obl., 27.vi.[1]975, Kasparjan” (under *Opius subcyclogaster*); 1 ♀ (ZISP), “[Russia:], Primorskij kraj, 20 km YuV Ussurijska, les, 5.viii. 1991, Belokobylskij”; 1 ♀ (ZISP), id., but nzap. “Kedrovaja Pad”, dubnjak, 22.vii.1979; 1 ♂ (ZISP), id., but Baradash-Levada, 2.ix.1978; 1 ♀ (ZISP), id., but Anisimovka, poljan, 12.vii.1984; 1 ♀ (ZISP), “[Russia:] Ilmenskij Zapoved, Tseljabinskoj obl., 17.vii.1950, Tobias”; 1 ♀ (ZISP), “**Kazachst[an**], Janvartsevo, prav., b. Urala, 31.viii.[1]949, Rubolph”; 1 ♀ (NWUX), “NW. **China**: **Shaanxi**, Xunyangba, Ningshan, c. 1300 m, 2.vi.2014, 33°33’N 108°32’E, Jiangli Tan, NWUX”; 1 ♀ (ZJUH), “[NE. China:] **Liaoning**, Shenyang, Dongling, 6.v.1994, Juxian Lou, No. 947532”; 2 ♀ (ZJUH), “[NE. China:] **Jilin**, Changbai Mts, 4.vii.1994, Juxuan Lou, Nos 951911 and 952014”; 2 ♀ (ZJUH), “[N. China:] **Henan**, Neixiang, Baotianman, 13 & 15.vii.1998 Yun Ma, Nos 986161 and 986801”; 1 ♀ (ZJUH), “[N. China:] Henan, Jigong Mts, 11.vii.1997, Xuexin Chen, No. 973737”; 2 ♀ (ZJUH), “[N. China:] **Hebei**, Xiaowutai Mts, Yangjiaping, 20.viii.2005, Min Shi, Hongying Zhang, Nos 200604624 and 200604804”; 1 ♀(ZJUH), “[SE. China]: **Fujian**, Chongan, Wuyi Mts, 5–10.vii.1989, Junhua He, No. 894760”; 1 ♀ (ZJUH), id., but 6.viii.1986, Jiashe Wang, No. 865476”; 2 ♀ (ZJUH), “[SE. China:] Fujian, Dehua, Daiyun Mts, 13 and 14.iv.2002, Yiping Wang, No. 20024716 and Jingxian Liu, No. 20024977”; 1 ♀ (ZJUH), “[SE. China:] Fujian, Dehua, Chishuizhen, 13.iv.2002, Zaifu Xu, No. 20025208”; 1 ♂ (ZJUH), “[SE. China:] Fujian, Liancheng, Tiaoxi, 18.viii.1988, Jian Huang, No. 20005629”; 2 ♂ (ZJUH), id., but Luochi, 23.viii.1988, Jian Huang, Nos 20005501 and 20005521”; 2 ♂ (ZJUH), “[SE. China:] Fujian, Nanping, Xiqinzhen, 21.ix.2002, Fangfang Li, Nos 20025524 and 20025551”; 1 ♀ 2 ♂ (ZJUH), “[SE. China:] Fujian, Shaxian, 15.ix.1980, Junhua He, No. 803805”; 1 ♀ 1 ♂ (ZJUH), id., but Yangfang, 1.vii.1981, Naiquan Lin, Nos 20044078 and 20044080”; 2 ♀ (ZJUH), “[SE. China:] Fujian, vi.1989, Zhishan Wu, Nos. 20009819 and 20009830”; 1 ♂ (ZJUH), “[SE. China:] Fujian, Yongan, Tianbaoyan, 15–18.vii.2001, Zaifu Xu, No. 20020238”; 5 ♀ (ZJUH), “[SE. China:] Fujian, Youxi, 15.v.1988, Qi Zheng, Nos 20005097, 20005106, 20005107, 20005122 and 20005148”; 2 ♀ (ZJUH), id., but Meixian, 15.x.1988, Changfu Lin, Nos 20005106 and 20005231”; 1 ♀ (ZJUH), “[S. China:] **Guangdong**, Fengkai, Heishiding, 15.viii.2003, Jujian Chen, No. 20048957”; 1 ♂ (ZJUH), “[S. China:] Guangdong, Guangzhou, 1.xi.1989, Junhua He, No. 896617”; 1 ♀ (ZJUH), “[S. China:] Guangdong, Huizhou, Xiangtou Mts, 11.v.2004, Zaifu Xu, No. 20053407”; 2 ♀ (ZJUH), “[S. China:] Guangdong, Yunan, Tongle Mts, 12–13.viii.2003, Zaifu Xu, Nos 20054397 and 20054613”; 3 ♀ 5 ♂ (ZJUH), “[S. China:] Guangdong, Yangchun, Baishui Waterfalls, 1.v.2002, Zaifu Xu, Nos 20028327, 20028352, 20028353, 20028371, 20028372, 20028383, 20028385 and 20028395”; 4 ♀ (ZJUH), id., but Baiyong, 5–6.v. 2002, Zaifu Xu, Nos 20028016, 20028022, 20028044 and 20028060; 2 ♀ (ZJUH), id., but Huatan, 34.v.2002, Zaifu Xu, Nos 20027570 and 20027811; 5 ♀ 1 ♂ (ZJUH), “[S. China:] Guangdong, Yangchun, Efengling Mts, 2.v.2002, Zaifu Xu, Nos 20028199, 20028221, 20028237, 20028238, 20028254 and 20028265”; 4 ♀ 1 ♂ (ZJUH), “[S. China:] Guangdong, Heyuan, Gui Mts, 18.v.2002, Zaifu Xu, Nos 20028572, 20028637, 20028657, 20028686 and 20028706”; 3 ♀ (ZJUH), “[S. China:] Guangdong, Shixing, Chebaling Mts, 21.viii.2003, Zaifu Xu, Nos 20051956, 20052375 and 20052443”; 3 ♀ (IZAS, RMNH) “[S. China:] **Hainan**, Tongshi, 340 m”, “3.iv.1960, Suofu Li”, “IOZ(E) 61743638”; 5 ♀ 1 ♂ (ZJUH), “[S. China:] Hainan, Yinggeling Mts, 18.x. 2007 and 24–25.v.2007, Jingxian Liu, Nos 200702620, 200702639, 200702754, 200702774, 200209739 and 200209997”; 1 ♀ (ZJUH), id., but Hongmao, 23–25.v.2007, Jie Zeng, No. 200804464; 1 ♀ (ZJUH), id., but 28.v.2007, Liqiong Weng, No. 200804194; 3 ♀ (ZJUH), “[S. China:] Hainan, Diaoluo Mts, 1–2.vi.2007 and 16–17.vii.2007, Jingxian Liu, Nos 200703899, 200703929 and 200802336”; 1 ♀ (ZJUH), “[S. China:] Hainan, Jianfengling Mts, 9–14.v.2007, Kuiyan Zhang, No. 200703651”; 4 ♀ (ZJUH), “[S. China:] Hainan, Wuzhi Mts, Shuimanxiang, 15–20.v.2007, Liqiong Weng, Nos 200803746, 200803755, 200803954 and 200803994”; 10 ♀ 7 ♂ (ZJUH), id., but 16–20.v.2007, 29.x.2007, Jingxian Liu, Nos 200703180, 200703261, 200703298, 200703385, 200710037, 200710040, 200710056, 200710091, 200710095, 200710114, 200710121, 200710129, 200710204, 200710205, 200710212, 200710282, 200710289 and 200710328”; 6 ♀ (ZJUH), id., but Shuimanxiong, 17–20.v.2007, Bin Xiao, Nos 200804666, 200804786, 200804793, 200804796, 200804814 and 200804857”; 1 ♀ (ZJUH), “[SW. China:] **Guangxi**, Fangcheng, Banba, 8.vi.2000, Hong Wu, No. 200100263”; 1 ♂ (ZJUH), “[SW. China:] Guangxi, Beiliu, 26.ix.1980, Youfu Zhong, No. 824470”; 1 ♂ (ZJUH), “[SW. China:] Guangxi, Daming Mts, Neichao,12.viii.2011, Chengjin Yan, No. 201100571”; 1 ♂ (ZJUH), “[SW. China:] Guangxi, Napo, Guinong Mts, 21.vi.2000, Hong Wu, No. 200100150”; 1 ♂ (ZJUH), “[SW. China:] Guangxi, Tianlin, Anjiaping, 29.v.1982, Junhua He, No. 821867”; 3 ♀ (ZJUH), “[SW. China:] Guangxi Botanical Garden, 30.x.2002, Naiquan Lin, Nos 20034981, 20034996 and 20035021”; 1 ♀ (ZJUH), “[SW. China:] **Sichuan**, Jiuzhaigou, 16.vii.1987, Gang Chen, No. 200012336”; 1 ♀ (ZJUH), “[SW. China:] **Yunnan**, Jinghong, 9.iv.1981, Junhua He, Nos 711675 and 811752”; 2 ♂ (ZJUH), “[SW. China:] Yunnan, Lancang, 20.iv.1981, Junhua He, Nos 814341 and 814358”; 1 ♂ (ZJUH), “[SW. China:] Yunnan, Mangshi, 9.v.1981, Junhua He, No. 813202”; 1 ♀ (ZJUH), “[SW. China:] Yunnan, Menghai, 17.iv.1981, Junhua He, No.811752”; 1 ♀ (ZJUH), “[SW. China:] Yunnan, Ruili, 4.v.1981, Junhua He, No. 815069”; 2 ♂ (ZJUH), id., but Mengxiu, 2–6.v.1981, Junhua He, Nos 813152 and 814057”; 2 ♀ (ZJUH), “[SW. China:] Yunnan, Tengchong, Jietouxiang, 11–12.vii.2006, Jie Zeng, Nos 20081636 and 20081839”; 1 ♀ (ZJUH), “[SW. China:] Yunnan, Youle Mts, 11.iv.1981, Junhua He, No. 811923”; 2 ♀ (ZJUH), “[SW. China:] Yunnan, Yuanjiang, 4.iv.1981, Junhua He, Nos 811414 and 811428”; 1 ♀ (ZJUH), “[E. China:] **Zhejiang**, Anji, Longwang Mts, 31.viii.1993, Xuexin Chen, No. 939738”; 1 ♀ (ZJUH), id., but 28.vii.1996, Hong Wu, No. 970389”; 1 ♀ (ZJUH), “[E. China:] Zhejiang, Gutian Mts, 1.viii.1990, Yun Ma, No. 906143”; 1 ♀ (ZJUH), “[E. China:] Zhejiang, Lin’an, Qingliangfeng Mts, 9.viii.2005, Hongying Zhang, No. 200607118”; 1 ♀ (ZJUH), “[E. China:] Zhejiang, Longquan, Fengyang Mts, 22–24.vii.1982, Qisheng Song, No. 826576”; 1 ♀ (ZJUH), “[E. China:] Zhejiang, Tianmu Mts, 21.vii.1987, Xuexin Chen, No.873064”; 1 ♀ (ZJUH), id., but 18.vi.1983, Yun Ma, No.831156; 2 ♀ (ZJUH), id., but Zuhua Shi, Nos 830471 and 830473; 1 ♀ 1 ♂ (ZJUH), id., but Junhua He, Nos 830703 and 830708; 1 ♀ (ZJUH), id., but 11.vi.1993, Yun Ma, No. 934354; 1 ♀ (ZJUH), id., but 20.vii.1987, Xuexin Chen, No. 872088; 2 ♀ (ZJUH), id., but 4.vi.1994, Xuexin Chen, Nos 941900 and 941912; 5 ♀ (ZJUH), id., but 1.vii.2000, Xuexin Chen, Nos 20032047, 20032048, 20032050, 20032059 and 20032079; 1 ♀ (ZJUH), id., but Chanyuan Temple, 16.v.1988, Xuexin Chen, No. 882029; 1 ♀ (ZJUH), id., but Xiaoming Lou, No. 883224; 5 ♀ (ZJUH), id., but 31.v.1998, Xuexin Chen, Nos 980067, 980149, 980158, 980504 and 980520; 1 ♀ (ZJUH), id. but Jinjing Fan, No. 884351; 2 ♀ 1 ♂ (ZJUH), id., but Laodian-Xianrending, 17–18.v.1988, Xuexin Chen, Nos 884383, 882587 and 891615; 1 ♀ (ZJUH), id., but Laodian, 13.vi.1998, Xuexin Chen, No. 980685; 2 ♀ (ZJUH), id., but Mingshui Zhao, Nos 20000806 and 20002334; 1 ♀ (ZJUH), id., but Sanmuping, 30.vii.1998, Mingshui Zhao, No. 999219; 1 ♀ (ZJUH), id., but Xianrending, 2–4.vi.1990, Yonggen Lou, No. 900124; 1 ♀ (ZJUH), id., but 3.vii.2000, Weidi Li, No. 200104179.

#### Comparative diagnosis.

As aptly indicated by its name the female lectotype of *Psyttalia cyclogaster* has the metasoma nearly circular because of the strongly transverse second and third tergites. Best to recognise by the scutellar subapical prominence, more or less developed smooth bump in front of anterior ocellus and pit behind stemmaticum, the laterally distinctly setose scutellum and the more or less distinctly micro-sculptured medio-posterior area of scutellum. According to the key by Fischer (1972) closely related to *Psyttalia nilotica* (Schmiedeknecht, 1900) from Egypt and Israel. However, the given differences (propodeum with bifurcate carina in *Psyttalia cyclogaster* and without in *Psyttalia nilotica*, and head mesosoma and base of metasoma mainly black in *Psyttalia cyclogaster* and reddish yellow in *Psyttalia nilotica*) are variable in the specimens examined and the possibility that *Psyttalia nilotica* is a pale southern form of *Psyttalia cyclogaster* should be considered. According to Fischer (1972, 1987) *Psyttalia nilotica* should have the precoxal sulcus narrow and the sulcus remains removed from the anterior border of the mesopleuron; this may allow a separation. In the key by Fischer (1987) *Psyttalia cyclogaster* runs to two S. African species: *Psyttalia vittator* (Brues, 1926) if bifurcate carina of propodeum is well developed and *Psyttalia prothoracalis* (Fischer, 1972) if carina is weakly developed or absent. Both species have the eye 1.5–1.6 times as long as temple in dorsal view (*vs* 2.5–5 times in *Psyttalia cyclogaster*) and, additionally, *Psyttalia prothoracalis* differs from both other species by the narrow, finely crenulate and long sinuate precoxal sulcus (*vs* medially wide, shorter and coarsely crenulate sulcus).

#### Description.

Redescribed ♀ from Shaanxi (Ningshan), length of body 3.9 mm, of fore wing 4.2 mm.

*Head*. Antenna with 36 segments and 1.1 times as long as fore wing; third segment as long as fourth segment, length of third, fourth and penultimate segments 3.3, 3.2 and 1.3 times their width, respectively (Figs 18, 23); length of maxillary palp 1.1 times height of head; length of eye in dorsal view 1.6 times temple (Fig. 20); temple in dorsal view shiny, smooth and with sparse setae; OOL: diameter of ocellus: POL = 18:7:10; area behind stemmaticum with a round depression and in front of anterior ocellus with a bump (Fig. 8); face largely smooth, with satin sheen and sparsely punctulate with a medio-longitudinal convexity dorsally and widened ventrally (Fig. 19); frons depressed behind antennal sockets, slightly shiny, glabrous and crenulate (Fig. 20); labrum depressed; clypeus nearly trapezoid, flat, and its ventral margin nearly straight and thin (Fig. 19); width of clypeus 1.9 times its maximum height and 0.4 times width of face; hypoclypeal depression wide and deep (Figs 19, 24); malar suture present, punctate between malar suture and clypeus (Fig. 24); mandible somewhat twisted and narrowed apically and normal basally, with narrow ventral carina (Fig. 24); occipital carina widely removed from hypostomal carina and dorsally absent; hypostomal carina narrow.

*Mesosoma*. Length of mesosoma 1.2 times its height; dorsal pronope absent (Fig. 20); pronotal side largely smooth, but anterior and posterior grooves present and coarsely crenulate (Fig. 15); epicnemial area crenulate dorsally; precoxal sulcus medially wide and coarsely crenulate, complete (Fig. 15); remainder of mesopleuron sparsely and finely punctate; pleural sulcus finely crenulate ventrally; mesosternal sulcus shallow and crenulate; postpectal carina absent; mesoscutum very shiny and glabrous (Fig. 16); notauli only anteriorly as pair of nearly smooth impressions and absent on disc; scutellar sulcus deep and with short crenulae, widened medially; scutellum distinctly convex and smooth, but medio-posteriorly longitudinally rugulose (Fig. 17); metanotum with a short longitudinal carina medially; surface of propodeum coarsely rugose and without an obvious medio-longitudinal carina (but bifurcate carina slightly indicated; Fig. 17) and anterior groove somewhat widened medially (Fig. 16).

*Wings*. Fore wing: 1-SR distinctly longer than wide and linear with 1-M (Fig. 14); pterostigma elongate triangular (Fig. 14); 1-R1 ending before wing apex and 1.5 times as long as pterostigma (Fig. 14); r long; r-m not tubular; r:3-SR:SR1 = 5:18:38; 2-SR:3-SR:r-m = 2:3:1; 1-M slightly curved near pterostigma and SR1 more or less straight; m-cu distinctly postfurcal and slightly curved; cu-a distinctly postfurcal and 1-CU1 widened; 1-CU1:2-CU1= 5:11; first subdiscal cell closed; CU1b short; only apex of M+CU1 sclerotized. Hind wing: 1-M straight; M+CU:1-M:1r-m = 14:13:10; cu-a straight; m-cu absent.

*Legs*. Length of femur, tibia and basitarsus of hind leg 4.2, 8.8 and 4.5 times as long as width, respectively (Fig. 25); hind femur and tibia with long setae.

*Metasoma*. Length of first tergite equal to its apical width, rather flat, its surface strongly and densely punctate-rugose (Fig. 17); second suture slightly indicated; second and following tergites smooth (except some superficial granulation), shiny and sparsely setose; combined length of second and third metasomal tergites 0.3 times total length of metasoma; length of setose part of ovipositor sheath 0.47 times fore wing, 3.5 times first tergite and 1.5 times hind tibia; hypopygium about 0.5 times as long as metasoma and distinctly acute apically (Fig. 26).

*Colour*. Black; head (including mandible) and propleuron yellowish brown, but teeth of mandible, stemmaticum and back of head dorsally black; scapus ventrally and tegula brown; pronotum ventrally, mesopleuron posteriorly and antero-dorsally, and metapleuron brown; palpi infuscate; humeral plate and legs yellowish, but tarsi brown; pterostigma and veins dark brown; laterally hypopygium brown and medially dark brown; fore wing membrane slightly infuscate.

*Variation*. Length of fore wing 2.4–4.2 mm; antenna of ♀ with 26(1), 28(1), 29(3), 34(1), 36(1), 37(1) and 38(1) segments; frons sculptured to often entirely smooth; hind femur 3.5–4.2 times as long as wide; first tergite 1.0–1.2 times as long as wide apically; setose part of ovipositor sheath 0.43–0.57 times as long as fore wing and 1.3–1.8 times hind tibia; second tergite entirely shiny granulate to (often entirely) smooth; head mainly black (except orbita) to nearly entirely orange or yellowish brown (except posteriorly), mesoscutum and mesopleuron largely black to entirely orange or yellowish brown; metasoma black to dark brown, sometimes first and second tergites brownish yellow or first tergite brown and second yellow or dark brown.

*Variation of types series*. The synonymy of *Coeloreuteus formosanus* Watanabe is based on photos of its holotype kindly supplied by Andrew Liston (SDEI); it is a pale specimen (with the head and the mesosoma mainly yellowish brown and the hind femur about 3.5 times as long as wide) having all the characteristics of *Psyttalia cyclogaster* as listed in the key. The only differences concern the paler head and mesosoma, smooth scutellum posteriorly and the more retracted (but equally long) hypopygium; these are considered insufficient for retaining it as valid species (both colour and sculpture are too variable in this species). *Rhogadopsis longicaudifera* Li & van Achterberg belongs also to this extreme form and is, therefore, also synonymized. *Psyttalia proclivis* (Papp) has first tergite of holotype only 1.1 times longer than its apical width (not 1.4 or 1.5 times as indicated by Papp (1981), Fischer (1989) and Tobias (1998)) and fits the diagnosis despite having the first tergite rather smooth. It shares this with *Psyttalia subcyclogaster* (Tobias) and both are rather small (length of body 2.0–2.7 mm and antenna with 28–29 segments). The holotype of *Psyttalia darasunica* (Tobias) differs mainly by the mainly black head and mesosoma, its rather small size, and having 29 antennal segments. In *Psyttalia cyclogastroides* (Tobias) the head and the mesosoma are partly brownish, the type specimens are larger and have 39 antennal segments. Finally, *Psyttalia extensa* Weng & Chen shares the micro-sculptured and setose medio-posterior area of scutellum (fig. 242 in Weng and Chen 2005), the frontal protuberance and the flattened medium-sized clypeus (Fig. 241, l.c.). The reported basally widened mandible is actually normal as shown on photographs of the holotype taken by Min-Lin Zheng (Fuzhou); it has only a ventro-basal carina.

#### Distribution.

France, Kazakhstan, Russia Far East (as *cyclogastroides*, *darasunicus* and *subcyclogaster*) Korea (as *proclivis*), China (Fujian (as *extensa*), *Guangdong, *Guangxi, *Hainan, *Henan, *Hebei, Hunan (as *longicaudifera*), Jilin (as *extensa*), *Liaoning, *Shaanxi, *Sichuan, Taiwan, *Yunnan, *Zhejiang), Japan (new record).

#### Biology.

Unknown.

### Psyttalia fletcheri

Taxon classificationAnimaliaHymenopteraBraconidae

(Silvestri, 1916)

Opius fletcheri Silvestri, 1916: 163–164; Wharton and Gilstrap 1983: 738.
Psyttalia (Psyttalia) fletcheri : Quicke et al. 1997: 25.
Psyttalia fletcheri : Wharton 1997: 23, 2009: 353; Fischer and Madl 2008: 1479–1480. Not Yao et al. (2008).

#### Comparative diagnosis.

*Psyttalia fletcheri* shares with the very similar *Psyttalia makii* and *Psyttalia incisi* the long vein r of fore wing (Fig. 28), the short temple (Fig. 32), vein 2-SR+M of fore wing distinctly widened (Fig. 28) and the antenna largely brownish yellow. Differs from *Psyttalia incisi* by the short vein 2-SR+M of fore wing (about twice as long as wide *vs* 3.5–4.0 times in *Psyttalia incisi*) and the strongly curved vein m-cu of fore wing (*vs* weakly curved or straight in *Psyttalia incisi*). Very similar to *Psyttalia makii*, but *Psyttalia fletcheri* has vein r of fore wing about as long as vein 2-SR (*vs* about 0.8 times vein 2-SR in *Psyttalia makii*) and vein 1-CU1 of fore wing at most 0.7 times as long as vein cu-a (*vs* about of equal length in *Psyttalia makii*).

#### Distribution.

Australia (Queensland), India, Indonesia, Malaysia, Réunion, Sri Lanka and Thailand. Introduced in Brazil, China (Taiwan), Fiji, Guam, Japan (Ryukyu Isl.), Philippines, Puerto Rico and U.S.A. (Hawaii, Florida).

#### Biology.

Parasitoid of Tephritidae: probably only of *Dacus* spp.; other reported hosts may be based on incorrect identification of the parasitoid (confusion with *Psyttalia incisi*) and/or host-relationship (Wharton and Gilstrap 1983). The male of *Psyttalia fletcheri* reported from mainland China (Guangdong) by Yao et al. (2008) reared from *Bactrocera dorsalis* (Hendel) is obviously misidentified. It is a species near *Psyttalia majocellata* sp. n., but differs by the short and widened vein 1-SR of the fore wing, the wider first subdiscal cell of fore wing, the dark brown pterostigma and the less sculptured frons.

### Psyttalia incisi

Taxon classificationAnimaliaHymenopteraBraconidae

(Silvestri, 1916)

Figs 28–32

Opius incisi Silvestri, 1916: 164–165; Beardsley 1961: 357; Wharton and Gilstrap 1983: 738; Ji et al. 2004: 144–145.
Psyttalia incisi : Wharton 1997: 23, 2009: 353.

#### Material.

4 ♀ 4 ♂ (RMNH, ZJUH), “S. **China**: **Fujian**, Fuzhou, reared in lab for release, 6.vi.2012, C. v. Achterberg, RMNH’12, *Psyttalia incisi* (Silvestri)”. The released reared specimens originate from locally collected stock (Ji et al. 2004).

#### Comparative diagnosis.

*Psyttalia incisi* shares with the very similar *Psyttalia makii* and *Psyttalia fletcheri* the long vein r of fore wing (Fig. 28) and the short temple (Fig. 32). *Psyttalia incisi* can be separated by having vein 2-SR+M of fore wing 3.5–4.0 times as long as wide (Fig. 28; *vs* about twice as long as wide in *Psyttalia makii* and *Psyttalia fletcheri*) and vein m-cu of fore wing weakly curved or straight (*vs* strongly curved in *Psyttalia makii* and *Psyttalia fletcheri*).

#### Distribution.

China (Fujian), India, Malaysia, Thailand, Philippines (Luzon). Introduced in U.S.A. (Hawaii, Florida), Mexico, Fiji, Guam and Australia (New South Wales, Queensland, Western Australia) (Yu et al. 2012).

#### Biology.

Parasitoid of Tephritidae: *Carpomyia vesuvuana* Costa, *Bactrocera carambolae* Drew & Hancock, *Bactrocera correcta* (Bezzi), *Bactrocera cucurbitae* (Coquillet), *Bactrocera dorsalis* (Hendel), *Bactrocera incisa* (Walker), *Bactrocera latifrons* (Hendel), *Bactrocera papayae* Drew & Hancock, *Bactrocera tuberculata* (Bezzi), *Ceratitis capitata* (Wiedemann) and *Dacus ciliatus* Loew.

#### Notes.

The series reared in the lab has either the basal half of pterostigma entirely dark brown and similar to its apical half (Fig. 28; males) or its basal half is yellow and contrasting with its dark brown apical half (females). The latter is considered to be typical (Wharton and Gilstrap 1983) but can be used only for females.

### Psyttalia latinervis

Taxon classificationAnimaliaHymenopteraBraconidae

Wu & van Achterberg sp. n.

http://zoobank.org/27F0CC72-A3A3-40D8-B672-D3F6AAA3BA60

Figs 33 , 34–43

#### Type material.

Holotype, ♂ (ZJUH), “[S. **China**:] **Hainan**, Bawangling Mts, 24–25.v.2007, Jingxian Liu, No. 200702714”.

#### Comparative diagnosis.

Easily recognizable species, because of the unique long, widened and slightly curved vein 1-CU1 of the fore wing (Fig. 35) in combination with the largely unsclerotized vein 1-SR+M, the widened but short vein 2-SR+M, and parallel veins m-cu and 1-M of the fore wing (Fig. 35).

#### Description.

Holotype, ♂, length of body 3.5 mm, of fore wing 2.8 mm.

*Head*. Antenna with 43 segments, bristly and rather adpressed setose and 1.7 times as long as fore wing; third segment 1.4 times as long as fourth segment, length of third, fourth and penultimate segments 3.0, 2.2 and 1.8 times their width, respectively (Fig. 43); length of maxillary palp 0.9 times height of head; length of eye in dorsal view 3.2 times temple (Fig. 40); temple shiny, smooth except for some punctures posteriorly and with sparse setae; OOL: diameter of ocellus: POL = 45:22:30; area behind stemmaticum reclivous (Fig. 40); face coarsely punctate with interspaces about equal to diameter of punctures and with satin sheen (Fig. 39); frons slightly depressed behind antennal sockets and in front of anterior ocellus, shiny, smooth and glabrous but laterally setose and punctulate (Fig. 40); labrum nearly flat; clypeus transverse, convex, and its ventral margin truncate and thin (Fig. 39); width of clypeus 3.5 times its maximum height and 0.8 times width of face; hypoclypeal depression wide and deep (Figs 39, 41); malar suture largely absent; malar space 0.4 times longer than basal width of mandible and area micro-sculptured (Fig. 41); mandible not twisted, apically moderately narrowed and with both teeth wide, normal basally and with narrow ventral carina (Fig. 41); occipital carina remains far removed from hypostomal carina and dorsally largely absent; hypostomal carina medium-sized ventrally.

*Mesosoma*. Length of mesosoma 1.2 times its height; pronope absent, only with groove; pronotal side largely smooth, but anterior and posterior grooves present and posteriorly with some crenulae (Fig. 36); propleuron flattened; epicnemial area smooth dorsally; precoxal sulcus only medially present and moderately crenulate (Fig. 36); remainder of mesopleuron smooth and shiny; pleural sulcus smooth ventrally; mesosternal sulcus shallow, narrow and finely crenulate; postpectal carina absent; mesoscutum very shiny and nearly entirely glabrous (Fig. 37); notauli only anteriorly as pair of partly finely crenulate impressions and absent on disc; scutellar sulcus deep and with 7 short crenulae, parallel-sided medially; scutellum slightly convex and smooth, only laterally sparsely setose (Fig. 37); metanotum with short longitudinal carina antero-medially and short carina posteriorly (Figs 37–38); surface of propodeum smooth, except for crenulae near reversed Y-shaped median carina and with short lateral crenulate groove above spiracle (Figs 37–38).

*Wings*. Fore wing: 1-SR as long as wide and linear with 1-M; pterostigma triangular and r not linear with postero-basal border (Fig. 34); 1-R1 ending at wing apex and 1.7 times as long as pterostigma; r linear with 3-SR and medium-sized; r-m and most of 1-SR+M unsclerotized; r:3-SR:SR1 = 5:29:56; 2-SR:3-SR:r-m = 15:29:7; 1-M straight and SR1 slightly curved; m-cu narrowly antefurcal and slightly curved, subparallel with 1-M (Fig. 35); 2-SR+M short and widened; cu-a short, vertical and far postfurcal; 1-CU1 curved and widened; 1-CU1:2-CU1= 15:24; first subdiscal cell widened apically and closed, CU1b medium-sized; only apex of M+CU1 sclerotized. Hind wing: 2-M slightly sinuate; M+CU:1-M:1r-m = 20:21:10; cu-a straight; m-cu and SR absent.

*Legs*. Length of femur, tibia and basitarsus of hind leg 4.2, 7.8 and 4.2 times as long as width, respectively (Fig. 42); hind femur with long setae.

*Metasoma*. Length of first tergite 1.4 times its apical width, convex medio-posteriorly, its surface largely smooth except some sculpture subposteriorly (Fig. 38), dorsal carinae strong in basal half of tergite and with depressed area below; second suture not indicated; basal depressions of second tergite deep and elliptical; second tergite 0.7 times as long as third tergite; second and following tergites smooth, shiny and sparsely setose; combined length of second and third metasomal tergites 0.35 times total length of metasoma.

*Colour*. Ivory or white; head dorsally (but stemmaticum black), scapus, pedicellus, V-shaped patch on mesoscutum, mesoscutum laterally, tegulae, scutellum largely and apical margin of third–seventh tergites yellow; remainder of antenna brown with apices of segments dark brown; scutellum posteriorly, metanotum and propodeum brownish; remainder of mesoscutum and of second–seventh tergites dorsally, pterostigma and veins dark brown; wing membrane subhyaline.

#### Distribution.

China (Hainan).

#### Biology.

Unknown.

#### Etymology.

From “latus” (Latin for “wide”) and “nervus” (Latin for “nerve, vein”) because of the widened vein 1-CU1 of the fore wing.

### Psyttalia majocellata

Taxon classificationAnimaliaHymenopteraBraconidae

Wu & van Achterberg sp. n.

http://zoobank.org/625ACC7F-A65D-4B4A-99D7-F611807B8EC6

Figs 44 , 45–52 , 53 , 54–64

#### Type material.

Holotype, ♀ (ZJUH), “[S. **China**:] **Hainan**, Bawangling Mts, 28.v.3.vi. 2007, Liqiong Weng, No. 200804217”. Paratypes (2 ♀ 2 ♂): 1 ♀ 2 ♂ (ZJUH, RMNH), id., but 9–10.vi.2007, Jingxian Liu, Nos 200703438, 200703465 and 201503525; 1 ♀ (ZJUH), “[SW. China:] **Guizhou**, Mayanghe river, 1–3.x.2007, Jingxian Liu, No. 200709564”.

#### Comparative diagnosis.

The new species runs in the key to the subgenus Psyttalia by Fischer (1987) to the Oriental *Psyttalia walkeri* (Muesebeck, 1931). The new species differs by having a short median carina on the propodeum, bifurcated medially and posterior half of propodeum with crenulae (Fig. 48; *vs* median carina long, bifurcated apically and posteriorly smooth in *Psyttalia walkeri*), POL equal to diameter of posterior ocellus (*vs* smaller), face and mesosoma similarly yellow (Fig. 46; *vs* face pale yellow, different from reddish yellow mesosoma), second tergite smooth (*vs* superficially granulate) and first tergite slightly longer than wide apically (Fig. 48; *vs* about 1.3 times). The new species can be easily confused with pale *Psyttalia carinata* (Thomson). The new species differs by having larger ocelli (OOL 1.2–1.7 times diameter of posterior ocellus and POL 0.8–1.0 times diameter of ocellus (Fig. 50) *vs* OOL 2.0–2.4 times diameter of posterior ocellus and POL slightly longer than diameter of ocellus in *Psyttalia carinata* (Fig. 8)), frons and vertex laterally punctate (*vs* largely smooth), vein 2-SR+M of fore wing 0.6–0.8 times as long as vein m-cu (*vs* about 0.4 times), second tergite half as long as third tergite (vs 0.8-0.9 times), first discal cell more transverse (*vs* transverse), base of hind tibia dark brown (*vs* brownish yellow) and distributed N. Oriental (*vs* Palaearctic). See note under *Psyttalia carinata* about a similar species from S. China.

#### Description.

Holotype, ♀, length of body 3.3 mm, of fore wing 3.2 mm.

*Head*. Antenna with 40+ segments (apical segments missing), bristly and rather erect setose and at least 1.3 times as long as fore wing; third segment 1.2 times as long as fourth segment, length of third and fourth penultimate segments 3.2 and 2.6 times their width, respectively (Fig. 44); maxillary palp 1.1 times as long as height of head; length of eye in dorsal view 3.9 times temple (Fig. 50); temple shiny, smooth except for some punctulation posteriorly and with sparse setae; OOL: diameter of ocellus: POL = 22:13:13; area behind stemmaticum reclivous (Fig. 50); face coarsely punctate with interspaces about equal to diameter of punctures and with satin sheen (Fig. 49); frons slightly depressed behind antennal sockets and with triangular depression between antennal sockets, shiny, smooth and glabrous but laterally (as vertex) setose and punctate (Fig. 50); labrum nearly flat; clypeus transverse, convex, punctate and its ventral margin truncate and thin (Fig. 49); width of clypeus 2.7 times its maximum height and 0.7 times width of face; hypoclypeal depression wide and deep (Fig. 49); malar suture largely absent; malar space 0.4 times longer than basal width of mandible and punctate; mandible not twisted, apically moderately narrowed and with both teeth wide, normal basally and with narrow ventral carina; occipital carina remains far removed from hypostomal carina and dorsally absent; hypostomal carina medium-sized ventrally.

*Mesosoma*. Length of mesosoma 1.4 times its height; pronope absent and only with groove; pronotal side largely smooth, but anterior and posterior grooves present, anteriorly and posteriorly with some crenulae (Fig. 46); propleuron flattened; epicnemial area smooth dorsally; precoxal sulcus moderately punctate-crenulate, absent posteriorly and nearly complete anteriorly (Fig. 46); remainder of mesopleuron smooth (except for band of fine punctures medially) and shiny; pleural sulcus smooth ventrally; mesosternal sulcus medium-sized and moderately crenulate; postpectal carina absent; mesoscutum very shiny and nearly entirely glabrous (Fig. 47); notauli only anteriorly as pair of partly finely crenulate impressions and absent on disc; scutellar sulcus deep and with 4 short crenulae, parallel-sided medially; scutellum slightly convex and smooth, only laterally sparsely setose (Fig. 47); metanotum with short longitudinal carina antero-medially and finely crenulate posteriorly (Fig. 47); surface of propodeum smooth, except for crenulae near reversed Y-shaped median carina (median carina part rather short), distinctly depressed posteriorly near triangular areola and with lateral crenulate groove above spiracle (Fig. 48).

*Wings*. Fore wing: 1-SR about 4 times longer than wide and linear with 1-M; pterostigma triangular and r linear with postero-basal border (Figs 45, 55); 1-R1 ending at wing apex and 1.7 times as long as pterostigma; r linear with 3-SR and medium-sized; r-m unsclerotized; 1-SR+M narrow and sclerotized; r:3-SR:SR1 = 2:9:16; 2-SR:3-SR:r-m = 23:45:13; 1-M straight and SR1 slightly curved; m-cu far antefurcal and straight, converging to 1-M (Fig. 45); 2-SR+M rather long and narrow (Fig. 55); cu-a medium-sized, oblique and far postfurcal; 1-CU1 straight and widened; 1-CU1:2-CU1= 15:24; first subdiscal cell widened apically and closed, CU1b medium-sized; only apex of M+CU1 sclerotized. Hind wing: 2-M slightly sinuate; M+CU:1-M:1r-m = 5:5:3; cu-a straight; m-cu and SR absent.

*Legs*. Length of femur, tibia and basitarsus of hind leg 3.5, 8.6 and 5.6 times as long as width, respectively (Fig. 42); hind femur with rather long setae.

*Metasoma*. Length of first tergite 1.1 times its apical width, convex medio-posteriorly, its surface largely finely rugose (Fig. 48), dorsal carinae strong in basal 0.7 of tergite and with depressed area below; second suture slightly indicated; basal depressions of second tergite deep and elliptical; second tergite 0.5 times as long as third tergite; second partly superficially coriaceous and following tergites smooth, shiny and sparsely setose; combined length of second and third metasomal tergites 0.25 times total length of metasoma; length of setose part of ovipositor sheath 0.47 times fore wing, as long as metasoma, 3.2 times first tergite, twice hind femur and 1.5 times hind tibia; hypopygium about 0.5 times as long as metasoma, distinctly acute apically and reaching apex of metasoma (Fig. 51).

*Colour*. Brownish yellow; stemmaticum black; antenna (except scapus and pedicellus but with dark patch on outer side, third segment darker than fourth one and apical segments becoming paler), ovipositor sheath, base of hind tibia and hind tarsus largely dark brown; tegulae pale yellow; palpi and base of legs ivory; pterostigma pale brown with margins darkened (Fig. 45) and veins brown; wing membrane subhyaline.

*Variation*. Length of fore wing 2.9–3.3 mm; antenna of ♀ with 37–44 segments and 1.4–1.5 times as long as fore wing; OOL 1.2–1.7 times diameter of posterior ocellus and POL 0.8–1.0 times diameter of ocellus; first tergite 1.1–1.3 times as long as its apical width (Figs 48, 59); hind femur 3.4–3.8 times as long as wide; setose part of ovipositor sheath 0.45–0.47 times as long as fore wing and 1.4–1.5 times hind tibia; second tergite more or less coriaceous; pterostigma of ♂ somewhat darker than of ♀ (Fig. 55); posterior areola of propodeum short (♀) or elongate triangular (♂) with long and rather short median carina, respectively (Figs 63–64); second–sixth tergites of ♂ partly dark brown and first tergite infuscate (Figs 53, 59); ♀ from Guizhou has base of hind tibia yellowish, basal half of antenna mainly brownish yellow (including third segment), propodeum more sculptured, antenna with 37 segments and second tergite almost entirely smooth. Males have mesoscutum only slightly darker brown laterally than medially, without distinct pattern (Fig. 58).

#### Distribution.

China (Hainan, Guizhou).

#### Biology.

Unknown.

#### Etymology.

From “major” (Latin for “larger”) and “ocellus” (Latin for “small eye”) because of the larger ocelli.

### Psyttalia makii

Taxon classificationAnimaliaHymenopteraBraconidae

(Sonan, 1932)

Opius makii Sonan, 1932: 68–69; Wharton and Gilstrap 1983: 739.
Psyttalia makii : Wharton, 1997: 23.

#### Comparative diagnosis.

Very similar to *Psyttalia fletcheri* because of the short vein 2-SR+M of fore wing (about twice as long as wide) and the strongly curved vein m-cu of fore wing. *Psyttalia makii* has vein r of fore wing about 0.8 times as long as vein 2-SR (about as long as vein 2-SR in *Psyttalia fletcheri*) and vein 1-CU1 of fore wing about as long as vein cu-a (at most 0.7 times as long as vein cu-a).

#### Distribution.

China (Taiwan, type locality); Indonesia (Java); Malaysia (Peninsular), Philippines (Mindanao); Thailand; U.S.A. (Hawaii, introduced but not retrieved).

#### Biology.

Parasitoid of Tephritidae: mainly reported from *Bactrocera* species (Yu et al. 2012).

### Psyttalia romani

Taxon classificationAnimaliaHymenopteraBraconidae

(Fahringer, 1935)

Figs 65 , 66–76

Opius (Marginopius) romani Fahringer, 1935: 9.
Opius romani : Fischer 1961: 13–15 (redescription), 1972: 346–347.
Opius (Psyttalia) romani : Tobias 1998: 613.
Psyttalia romani : Tobias 2000: 12; Chen and Weng 2005: 152.

#### Material.

2 ♀ (ZISP), “[**Russia**:], Amurskaja oblast, s. Novorossijka, r. Selemdzha, 1–10.viii.1966, D. Kasparjan”; 1 ♀ (ZISP), “[Russia:], Primorskij kraj, okr. Nachodki, dubnjak kustarnik, 20.viii.1985, Belokobylskij”; 1 ♀ (ZISP), id., but Baradazh-Levada, 2.ix.1978, “*Opius romani* Fahr., det. Tobias 1994”; 1 ♀ (ZJUH), “[NW. **China**:] Shaanxi, Dasanguan, 4.ix.1999, Ping Cai, No. 200011724”.

#### Comparative diagnosis.

In the East Palaearctic region the only similar *Psyttalia* species known is *Psyttalia sakhalinica* (Tobias) because of the similar gradually narrowed head in dorsal view (Figs 72, 84). *Psyttalia romani* differs by having mesosoma orange brown, contrasting with mainly black metasoma (*vs* meso- and metasoma mainly black or dark brown in *Psyttalia sakhalinica*), hind femur 2.9–3.3 times as long as wide (*vs* 3.5–3.9 times), fore wing distinctly infuscate (*vs* slightly infuscate) and legs yellowish brown (*vs* brownish yellow).

#### Description.

Redescribed after ♀ from Novorossijka, length of body 4.4 mm, of fore wing 4.4 mm.

*Head*. Antenna with 47 segments, bristly and erect setose and 1.4 times as long as fore wing; third segment 1.6 times as long as fourth segment, length of third, fourth and penultimate segments 3.4, 2.2 and 1.9 times their width, respectively (Figs 70, 75–76); length of maxillary palp equal to height of head; length of eye in dorsal view 2.2 times temple (Fig. 72); temple in dorsal view shiny, smooth and with sparse setae; OOL: diameter of ocellus: POL = 14:5:8; area behind stemmaticum flat (Fig. 72); face coarsely punctate with most interspaces wider than diameter of punctures, shiny and smooth medio-longitudinal convexity dorsally and widened ventrally (Fig. 71); frons slightly depressed behind antennal sockets and in front of anterior ocellus slightly impressed, shiny, smooth and glabrous but laterally with few setae (Fig. 72); labrum slightly depressed; clypeus transverse, convex, with some coarse punctures and its ventral margin protruding, with fringe of long setae and rather thin (Fig. 71); width of clypeus 3.4 times its maximum height and 0.7 times width of face; hypoclypeal depression wide and deep (Figs 67, 71); malar suture indistinct except for deep depression near eye, sparsely punctate-rugose between malar suture and clypeus (Fig. 74); mandible not twisted, apically moderately narrowed and with both teeth wide; mandible normal basally and with narrow ventral carina (Fig. 74); occipital carina remains far removed from hypostomal carina and dorsally largely absent; hypostomal carina rather wide ventrally.

*Mesosoma*. Length of mesosoma 1.2 times its height; dorsal pronope absent; pronotal side largely smooth, but posteriorly grooves with some crenulae (Fig. 67); propleuron flattened; epicnemial area smooth dorsally; precoxal sulcus anteriorly and medially rather narrowly crenulate, absent posteriorly (Fig. 67); remainder of mesopleuron smooth and shiny except for some crenulae dorsally; pleural sulcus smooth ventrally except for a few short crenulae; mesosternal sulcus deep, narrow and finely crenulate; postpectal carina absent; mesoscutum very shiny and glabrous (Fig. 68); notauli only anteriorly as smooth impressions and absent on disc; scutellar sulcus deep and with 5 short crenulae, parallel-sided medially; scutellum slightly convex and smooth, but laterally sparsely punctulate and setose (Fig. 68); metanotum with short longitudinal carina antero-medially and finely crenulate posteriorly; surface of propodeum smooth dorsally but posteriorly and area near distinct and reversed Y-shaped median carina rugose (Fig. 68), lateral grooves shallow and irregularly rugose.

*Wings*. Fore wing: 1-SR distinctly longer than wide and linear with 1-M (Fig. 66); pterostigma triangular and r linear with postero-basal border (Fig. 66); 1-R1 ending at wing apex and 1.6 times as long as pterostigma; r linear with 3-SR and medium-sized; r-m not tubular; r:3-SR:SR1 = 10:40:73; 2-SR:3-SR:r-m = 22:40:13; 1-M and SR1 slightly curved; m-cu distinctly antefurcal, converging to 1-M posteriorly and slightly curved, 2-SR+M rather widened (as apex of M+CU1: Fig. 66); cu-a distinctly postfurcal and 1-CU1 widened; 1-CU1:2-CU1= 3:22; first subdiscal cell closed; CU1b medium-sized; only apical fifth of M+CU1 sclerotized. Hind wing: 1-M straight; M+CU:1-M:1r-m = 22:23:15; cu-a straight; m-cu absent; SR slightly indicated apically.

*Legs*. Length of femur, tibia and basitarsus of hind leg 2.9, 6.8 and 4.2 times as long as width, respectively (Fig. 73); hind femur with long setae, tarsus and tibia densely setose (Fig. 73).

*Metasoma*. Length of first tergite equal to its apical width, convex medio-posteriorly, its surface largely coarsely rugose (Fig. 69), dorsal carinae strong in its basal half and with depressed area below; second suture slightly indicated; pair of basal depressions of second tergite large and tergite 0.9 times as long as third tergite; second and following tergites smooth, shiny and sparsely setose; combined length of second and third metasomal tergites 0.25 times total length of metasoma; length of setose part of ovipositor sheath 0.56 times fore wing, 4.9 times first tergite, 2.4 times hind femur and 1.7 times hind tibia; hypopygium 0.6 times as long as metasoma, distinctly acute apically and surpassing apex of metasoma (Fig. 73).

*Colour*. Orange brown, but stemmaticum and metasoma (except mainly reddish brown first tergite, lateral patches of sternites and tergites and hypopygium dorsally brown), tegulum pale yellowish and humeral plate infuscate; palpi, scapus and pedicellus ventrally and legs yellowish brown, but telotarsi infuscate; pterostigma and veins dark brown; fore wing membrane distinctly infuscate, especially near veins.

*Variation*. Length of fore wing 4.4–4.7 mm; antenna of ♀ with 47 segments; dorsal pronope absent or present as small round pit; vein 3-SR of fore wing 1.4–1.8 times as long as vein 2-SR; hind femur 2.9–3.2 times as long as wide; setose part of ovipositor sheath 0.46–0.56 times as long as fore wing and 1.5–1.7 times hind tibia.

#### Distribution.

China (Gansu, *Shaanxi), Russia Far East, Korea.

#### Biology.

Unknown.

### Psyttalia sakhalinica

Taxon classificationAnimaliaHymenopteraBraconidae

(Tobias, 1998)

Figs 77 , 78–88

Opius (Psyttalia) sakhalinicus Tobias, 1998: 612.
Psyttalia sakhalinica : Tobias 2000: 12.

#### Type material.

Holotype, ♀ (ZISP), “[**Russia**], 10 km z Anivy, smles, Sachalin, 15.vii.[1]981, Belokobylskij”, “*Opius sakhalinicus* sp. n., det. Tobias, [19]95”; “Holotypus *Opius sakhalinicus* Tobias”.

#### Additional material.

1 ♀ (ZISP) “[Russia], o. Kunamir, Yu.-Kurilsk, r. lesky, 19.viii.1989, A. Lelej”, “*Psyttalia sakhalinicus* Tob., Tobias det. 2001”.

#### Comparative diagnosis.

See *Psyttalia romani* (Fahringer).

#### Description.

Holotype, ♀, length of body 4.6 mm, of fore wing 4.8 mm.

*Head*. Antenna with 45 segments, bristly and erect setose and 1.3 times as long as fore wing; third segment 1.4 times as long as fourth segment, length of third, fourth and penultimate segments 2.8, 2.0 and 2.3 times their width, respectively (Figs 82, 87–88); length of maxillary palp 1.3 times height of head; length of eye in dorsal view 2.5 times temple (Fig. 84); temple in dorsal view shiny, smooth and with sparse setae; OOL: diameter of ocellus: POL = 9:5:6; area behind stemmaticum flat (Fig. 84); face coarsely punctate with interspaces about equal to diameter of punctures, with satin sheen and sparsely punctulate with a medio-longitudinal convexity dorsally and widened ventrally (Fig. 83); frons slightly depressed behind antennal sockets and in front of anterior ocellus, shiny, smooth and glabrous but laterally setose and punctulate (Fig. 84); labrum slightly depressed; clypeus transverse, convex, and its ventral margin concave, obtuse and thick (Fig. 83); width of clypeus 5.0 times its maximum height and 0.7 times width of face; hypoclypeal depression wide and deep (Figs 79, 83); malar suture indistinct except for deep depression near eye, punctate-rugose between malar suture and clypeus (Fig. 86); mandible not twisted, apically moderately narrowed and with both teeth wide; mandible normal basally and with narrow ventral carina (Fig. 86); occipital carina remains far removed from hypostomal carina and dorsally largely absent; hypostomal carina rather wide ventrally.

*Mesosoma*. Length of mesosoma 1.2 times its height; dorsal pronope small, round; pronotal side largely smooth, but anterior and posterior grooves present and largely smooth (Fig. 79); propleuron flattened; epicnemial area smooth dorsally; precoxal sulcus medially medium-sized and only medially distinctly crenulate, absent posteriorly (Fig. 79); remainder of mesopleuron smooth and shiny; pleural sulcus smooth ventrally; mesosternal sulcus deep, narrow and finely crenulate; postpectal carina absent; mesoscutum very shiny and glabrous (Fig. 80); notauli only anteriorly as pair of nearly smooth impressions and absent on disc; scutellar sulcus deep and with 4 short crenulae, parallel-sided medially; scutellum slightly convex and smooth, but laterally sparsely punctulate and setose (Fig. 80); metanotum without a longitudinal carina medially and finely crenulate posteriorly; surface of propodeum smooth except for rugose area near distinct and reversed Y-shaped median carina (Fig. 80), lateral grooves shallow and irregularly rugose and anterior groove somewhat widened medially (Fig. 80).

*Wings*. Fore wing: 1-SR distinctly longer than wide and linear with 1-M (Fig. 78); pterostigma triangular and r linear with postero-basal border (Fig. 78); 1-R1 ending at wing apex and 1.4 times as long as pterostigma (Fig. 78); r linear with 3-SR and medium-sized; r-m not tubular; r:3-SR:SR1 = 5:22:44; 2-SR:3-SR:r-m = 15:22:7; 1-M and SR1 straight; m-cu distinctly antefurcal and slightly curved, 2-M+CU1 rather widened (as apex of M+CU1: Fig. 78); cu-a distinctly postfurcal and 1-CU1 widened; 1-CU1:2-CU1 = 2:11; first subdiscal cell closed; CU1b medium-sized; only apex of M+CU1 sclerotized. Hind wing: 1-M straight; M+CU:1-M:1r-m = 30:24:11; cu-a straight; m-cu absent; SR slightly indicated.

*Legs*. Length of femur, tibia and basitarsus of hind leg 3.9, 8.3 and 5.4 times as long as width, respectively (Fig. 85); hind femur and tibia with long setae.

*Metasoma*. Length of first tergite 1.1 times to its apical width, convex medio-posteriorly, its surface strongly and densely rugose (Fig. 81), dorsal carinae strong in its basal half and with depressed area below; second suture slightly indicated; basal depressions of second tergite large and tergite 0.9 times as long as third tergite; second and following tergites smooth, shiny and sparsely setose; combined length of second and third metasomal tergites 0.25 times total length of metasoma; length of setose part of ovipositor sheath 0.53 times fore wing, 3.8 times first tergite, 2.3 times hind femur and 1.7 times hind tibia; hypopygium about 0.5 times as long as metasoma, distinctly acute apically and reaching apex of metasoma (Fig. 85).

*Colour*. Black, but head (except dark brown frons and vertex but excluding orbita) and propleuron, propleuron ventrally, tegulae, scapus ventrally, sternites (except medially) and second-seventh tergites laterally largely orange brown; palpi, mandible (but teeth dark brown) and legs brownish yellow, but apical half of tarsi infuscate; metasoma apically, remainder of propleuron and mesopleuron anteriorly dark brown; pterostigma and veins dark brown; fore wing membrane slightly infuscate.

*Variation*. Length of fore wing 4.8–5.0 mm; antenna of ♀ with 44–45 segments; first tergite 1.0–1.1 times as long as its apical width, more or less flattened; precoxal sulcus nearly smooth to distinctly crenulate medially; face punctate to densely punctate-rugose; hind femur 3.5–3.9 times as long as wide; setose part of ovipositor sheath 0.51–0.53 times as long as fore wing and 1.6–1.7 times hind tibia; second tergite black or orange brown anteriorly.

#### Distribution.

Russia Far East.

#### Biology.

Unknown.

### Psyttalia spectabilis

Taxon classificationAnimaliaHymenopteraBraconidae

van Achterberg sp. n.

http://zoobank.org/7F3B01AA-ADD9-4EA0-908B-52654CA14FB5

Figs 89 , 90–99

#### Material.

Holotype, ♀ (RMNH), “Museum Leiden, **Japan**[: Honshu], Gaga Spa-Zaô, Miyagi Pref., 31.vii.1981, A. Takasu”. Paratype: 1 ♀ (RMNH) with same data as holotype.

#### Comparative diagnosis.

The new species runs in the keys to Palaearctic Opiinae by Fischer (1972) to *Diachasma mysticum* (= *Rhogadopsis mystica* (Fischer, 1963) comb. n.) from Japan. It differs from *Rhogadopsis mystica* by having the head and mesosoma (except propodeum and metapleuron) brownish yellow (*vs* head, except clypeus, and mesosoma black in *Rhogadopsis mystica*), vein CU1b of fore wing much shorter than vein 3-CU1 (Fig. 90; *vs* vein CU1b about as long as vein 3-CU1); pterostigma distinctly triangular (Fig. 90; *vs* elongate); medio-posterior depression of mesoscutum absent (*vs* present); vein r of fore wing continuous with vein 3-SR (Fig. 90; *vs* vein r of fore wing rather angled with vein 3-SR); vein SR1 of fore wing about 1.8 times vein 3-SR (Fig. 90; *vs* vein SR1 of fore wing about 2.7 times vein 3-SR) and length of body 5–6 mm (*vs* about 3 mm). In the key by Fischer (1987) the new species runs to the Oriental *Psyttalia walkeri* (Muesebeck). The new species differs by having lateral crenulate grooves on the propodeum (Fig. 93; *vs* absent and instead with carina in *Psyttalia walkeri*), propodeum and first–fifth tergites largely black (*vs* reddish yellow or partly infuscate), hind tibia (except ventrally) and tarsus dark brown, contrasting with ivory hind femur (Fig. 99; *vs* hind femur, tibia and tarsus similar pale yellow), pterostigma dark brown (*vs* pale yellow), length of body 5–6 mm (*vs* 2–3 mm) and vein 2-CU1 of fore wing at same level as vein M+CU1 (Fig. 90; *vs* vein 2-CU1 distinctly below level of vein M+CU1).

#### Description.

Holotype, ♀, length of body 5.6 mm, of fore wing 5.2 mm.

*Head*. Antenna with 52+ segments (its apex missing), bristly and erect setose and 1.4 times as long as fore wing; third segment 1.2 times as long as fourth segment, length of third and fourth segments 2.6 and 2.1 times their width, respectively (Figs 97–98); length of maxillary palp 1.2 times height of head; length of eye in dorsal view 4.6 times temple (Fig. 96); temple in dorsal view shiny, largely smooth and with sparse punctures; OOL: diameter of ocellus: POL = 9:5:4; area behind stemmaticum with groove, widened laterally (Fig. 96); face moderately punctate with interspaces wider than diameter of punctures, except submedially, shiny and medio-longitudinal convexity mainly smooth and ventrally widened (Fig. 95); frons moderately depressed behind antennal sockets, shiny, rugose and glabrous but laterally setose and punctulate, in front of anterior ocellus with narrow groove and narrow smooth ridge (Fig. 96); labrum flat; clypeus transverse, convex, coarsely punctate and its ventral margin slightly convex and thin (Fig. 95); width of clypeus 4.0 times its maximum height and 0.8 times width of face; hypoclypeal depression wide and deep (Figs 91, 95); malar space narrow (Fig. 95); malar suture indistinct except for deep depression near eye, between malar suture and clypeus punctate; mandible not twisted, apically moderately narrowed, punctate and with both teeth wide; mandible normal basally and with narrow ventral carina (Fig. 91); occipital carina remains far removed from hypostomal carina and dorsally largely absent; hypostomal carina rather wide ventrally.

*Mesosoma*. Length of mesosoma 1.3 times its height; dorsal pronope small, round; pronotal side largely smooth, but anterior and posterior grooves present and coarsely crenulate (Fig. 91); propleuron flattened; epicnemial area smooth dorsally; precoxal sulcus medially medium-sized and only medially distinctly crenulate, absent anteriorly and posteriorly (Fig. 91); remainder of mesopleuron smooth and shiny; pleural sulcus very finely crenulate ventrally; mesosternal sulcus deep, narrow and finely crenulate; postpectal carina absent; mesoscutum shiny and glabrous (Fig. 92); notauli only anteriorly as pair of nearly smooth impressions and absent on disc, but notaulic courses indicated by setae and punctulation; scutellar sulcus deep and with 5 long crenulae, parallel-sided medially; scutellum rather convex and smooth, but laterally sparsely punctulate and setose (Fig. 92); metanotum with a short medio-longitudinal carina anteriorly and its posterior face finely crenulate; surface of propodeum smooth except for crenulate grooves near distinct and reversed Y-shaped median carina (Fig. 93), lateral grooves deep and coarsely regularly crenulate, and anterior groove somewhat widened medially (Fig. 93).

*Wings*. Fore wing: 1-SR longer than wide and slightly angled with 1-M (Fig. 90); pterostigma wide triangular and r nearly linear with postero-basal border (Fig. 90); 1-R1 ending at wing apex and 1.3 times as long as pterostigma (Fig. 90); r nearly linear with 3-SR and medium-sized; r-m not tubular; r:3-SR:SR1 = 5:20:42; 2-SR:3-SR:r-m = 13:20:6; 1-M straight; SR1 distinctly curved; m-cu distinctly antefurcal, subparallel with 1-M and straight, 2-SR+M slender (as apex of M+CU1: Fig. 90); cu-a distinctly postfurcal and 1-CU1 widened; 1-CU1:2-CU1 = 5:31; first subdiscal cell closed; CU1b medium-sized; only apex of M+CU1 sclerotized. Hind wing: 1-M straight; M+CU:1-M:1r-m = 30:35:13; cu-a straight; m-cu absent; SR entirely absent.

*Legs*. Length of femur, tibia and basitarsus of hind leg 3.4, 8.2 and 4.9 times as long as width, respectively (Fig. 99); hind femur and tibia with long setae and densely setose.

*Metasoma*. Length of first tergite 1.1 times to its apical width, convex medio-posteriorly, convexity surrounded by crenulate groove, its surface densely punctate-rugose (Fig. 93), dorsal carinae strong in its basal half and with depressed area below; second suture slightly indicated; basal depressions of second tergite medium-sized and tergite 0.7 times as long as third tergite, both smooth (except some punctulation) and largely setose; following tergites smooth, shiny and sparsely setose; combined length of second and third metasomal tergites 0.26 times total length of metasoma; sixth tergite membranous medio-posteriorly; length of setose part of ovipositor sheath 0.46 times fore wing, 2.9 times first tergite, 2.0 times hind femur, 1.4 times hind tibia and 0.9 times metasoma; hypopygium 0.35 times as long as metasoma, acute apically and reaching apex of metasoma (Fig. 94).

*Colour*. Brownish yellow; propodeum, first tergite, second tergite except laterally, third tergite except posteriorly, fourth and fifth tergites (but anteriorly and posteriorly brownish) black; metapleuron chestnut brown; palpi, legs (but hind tibia and tarsus mainly dark brown) and remainder of metasoma ivory; tegulae pale yellowish; antenna (but scapus and pedicellus mainly yellow), pterostigma and veins dark brown; fore wing membrane subhyaline.

*Variation*. Paratype: length of fore wing 4.3 mm; antenna with 52 segments; first tergite 1.1 times as long as its apical width and only superficially punctate medially; hind femur 3.8 times as long as wide; setose part of ovipositor sheath 0.47 times as long as fore wing and 1.5 times hind tibia; hind tibia ivory ventrally and propodeum chestnut brown.

#### Distribution.

Japan.

#### Biology.

Unknown.

#### Etymology.

The name refers to the showy combination of colours of this species: “spectabilis” is Latin for “showy, notable”.

#### Notes.

*Rhogadopsis mystica* (Fischer, 1963) comb. n. was originally described in the genus *Opius* Wesmael and up to now only known of the male holotype. It was later included in *Diachasma* Foerster, 1863, by Fischer (1972). The latter is an obvious misfit because the clypeus is truncate ventrally (*vs* convex in *Diachasma*) and it has a distinct hypoclypeal depression below it (*vs* absent or as a narrow slit in *Diachasma*), vein 3-SR of fore wing longer than vein 2-SR and vein m-cu of hind wing absent (according to the original description veins 2-SR and 3-SR equal, but in the figured fore wing 3-SR 1.2 times longer than 2-SR; *vs* in *Diachasma* vein 3-SR usually shorter than vein 2-SR and if subequal then vein m-cu of hind wing at least present as a distinctly pigmented trace). Tobias (1998) included it in the subgenus Aulonotus Ashmead of *Opius* Wesmael. *Aulonotus* Ashmead is a synonym of *Xynobius* Foerster, 1863 (Li et al. 2013), but it is unlikely that it belongs there because the dorsal carinae are weakly developed, the marginal cell of the hind wing is wide and vein 3-SR of fore wing slightly longer than vein 2-SR (Fischer 1963). According to the original description vein m-cu of fore wing is distinctly curved and gradually merging into vein 2-CU1, vein 1r-m of hind wing is weakly oblique and 0.7 times as long as vein 1-M, hind wing comparatively wide and medio-longitudinal carina of propodeum present anteriorly, what agrees well with the definition of *Rhogadopsis* Brèthes, 1913 (Li et al. 2013). It can be separated from other *Rhogadopsis* species by its complete notauli combined with the antefurcal vein m-cu, short vein 1-SR and distally widened first subdiscal cell of the fore wing.

### Excluded species

#### Rhogadopsis mediocarinata

Taxon classificationAnimaliaHymenopteraBraconidae

(Fischer, 1963) comb. n.

Figs 100 , 101–110

Opius mediocarinatus Fischer, 1963: 297 (examined).
Opius (Lissosema) mediocarinatus : Fischer 1972: 360–361.
Opius (Psyttalia) mediocarinatus : Tobias 1998: 611.
Psyttalia mediocarinata : Tobias 2000: 12.
Opius (Lissosema) longurius Chen & Weng, 2005: 99–101, 197 (examined). **Syn. n.**
Rhogadopsis longuria : Li et al. 2013: 154–157 (redescription).
Opius (Psyttalia) vacuus Tobias, 1998: 612 (examined). **Syn. n.**
Opius vacuus : Tobias 2000: 15.

##### Type material.

Holotype of *Opius longurius*, ♀ (FAFU), “[**China**:] **Fujian**, Wuyi Mt., Sangang, 30.vi.1988, Zhang Xia-bin”. Holotype of *Opius vacuus*, ♀ (ZISP), “[**Russia**], Primorskij kraj, Spassk, les, poljany, 19.viii.1991, Belokobylskij”, “*Opius vacuus* sp. n., det. Tobias ‘95”, “Holotypus *Opius vacuus* Tobias”. Paratype of *Opius mediocarinatus*. ♀ (MTMA) from Japan (Honshu: Kamikochi) examined.

##### Comparative diagnosis.

The combination of lacking the medio-posterior depression of the mesoscutum (Fig. 103) and the slender first metasomal tergite with a long median carina (Fig. 104) makes this species easy to separate from all other species of *Rhogadopsis* in China.

##### Distribution.

China (Fujian (as *longurius*), Hunan (as *longuria*), *Shaanxi), Russia Far East, Japan, Korea. The record from Spain (Avinent and Jiménez 1987) needs reconfirmation.

##### Biology.

Unknown.

##### Notes.

The inclusion of *Opius mediocarinatus* Fischer from Japan in *Psyttalia* by Tobias (1998, 2000) is an obvious misfit; it is also excluded by Wharton (2009). It has a short (hardly protruding) ovipositor (Fig. 100), vein m-cu of fore wing 0.65 times as long as vein 1-M, vein m-cu of fore wing angled with vein 2-CU1, and a normal second tergite and hypopygium. It belongs to the genus *Rhogadopsis* Brèthes, 1913, as defined by Li et al. (2013) and is one of the easier identifiable species of the genus because of the shape and sculpture of the first tergite.

The holotype of *Opius vacuus* is a very typical *Rhogadopsis mediocarinata* because of the reduced posterior groove of the pronotal side, the striped mesoscutum and the elongate first metasomal tergite with the distinct median carina. Vein 1r-m of the hind wing is rather short (0.55 times as long as vein 1-M), but obviously this vein is rather variable in this species and vein 1-M of hind wing has a weak bend subapically.

### Addendum

*Psyttoma latilabris* (Chen & Weng, 2005) is similar to a *Psyttalia* species because of the enlarged and apically acute hypopygium of ♀, but differs because of the medially protruding scutellum (above level of mesoscutum), the narrow hind wing with short vein 1r-m, the wide face and hind femur (length about 3.0 times its width). In ZJUH is material of this species present from *Xinjiang province (NW. China: 1 ♀ 1 ♂, Shihezi, 12.vii.2001, Hongying Hu, Nos 200304217 and 20036001; 1 ♂, Wulumuqi, 3.viii.2001, Hongying Hu, No. 20036044; 2 ♂ Badanbohu, 7.viii.2001, Hongying Hu, Nos 20036055 and 20036060; 2 ♂, Nongqishi, 12.vii.2001, Hongying Hu, No. 20036093). To date, this species is known from Shandong and Hubei provinces (Li et al. 2012).

## Supplementary Material

XML Treatment for Psyttalia

XML Treatment for Psyttalia carinata

XML Treatment for Psyttalia cyclogaster

XML Treatment for Psyttalia fletcheri

XML Treatment for Psyttalia incisi

XML Treatment for Psyttalia latinervis

XML Treatment for Psyttalia majocellata

XML Treatment for Psyttalia makii

XML Treatment for Psyttalia romani

XML Treatment for Psyttalia sakhalinica

XML Treatment for Psyttalia spectabilis

XML Treatment for Rhogadopsis mediocarinata
